# Supplementary material for: Synthesis and Evaluation of NF-κB Inhibitory Activity of Mollugin Derivatives
Source: Molecules. 2022 Nov 16;27(22):7925. doi: 10.3390/molecules27227925 (PMC9695821; doi:10.3390/molecules27227925)
Supplement: Supplementary file 1 [file molecules-27-07925-s001.zip › molecules-1971500-supplementary.pdf]

molecules

Article

Synthesis and Evaluation of NF-κB Inhibitory Activity of Mollugin Derivatives

Lin-Hao Zhang [1], Ming-Yue Li [1], Da-Yuan Wang [1], Xue-Jun Jin [1], Fen-Er Chen [1,2,]* and Hu-Ri Piao [1,]*

Content

The spectrum of ^{1}H NMR, ^{13}C NMR and HRMS of compounds 4a-I …………….. 2-15

The spectrum of ^{1}H NMR, ^{13}C NMR and HRMS of compounds 6a-k…………….. 15-32

The spectrum of ^{1}H NMR, ^{13}C NMR and HRMS of compounds 8a-c …………….. 32-36

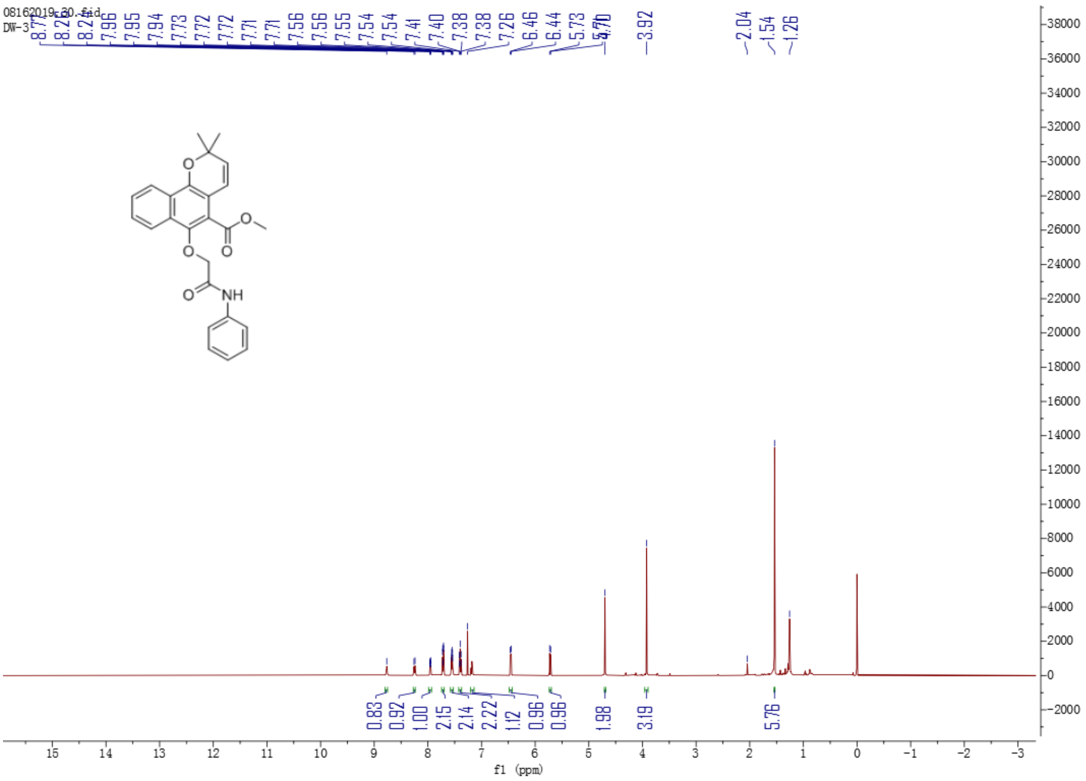

Figure S1. ^{1}H-NMR (300 MHz, $CDCl_3$) spectrum of Mollugin derivative **4**[a]

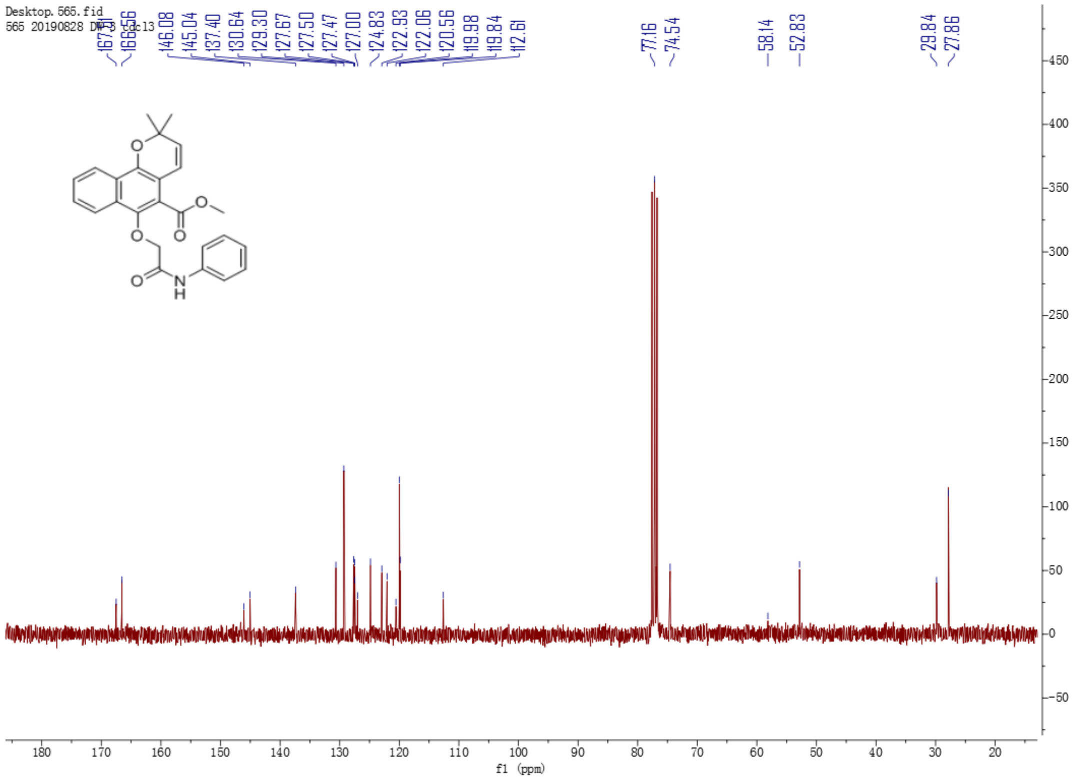

Figure S2. ^{13}C NMR (125 MHz, $CDCl_3$) spectrum of Mollugin derivative **4a**

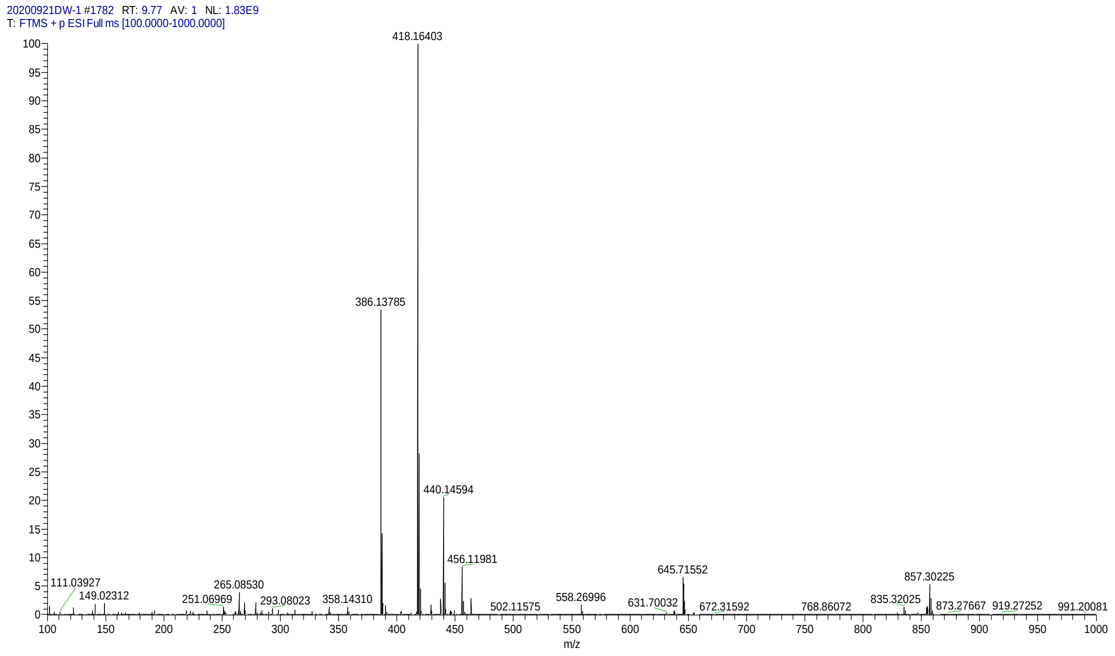

Figure S3. HRMS of Mollugin derivative **4a**

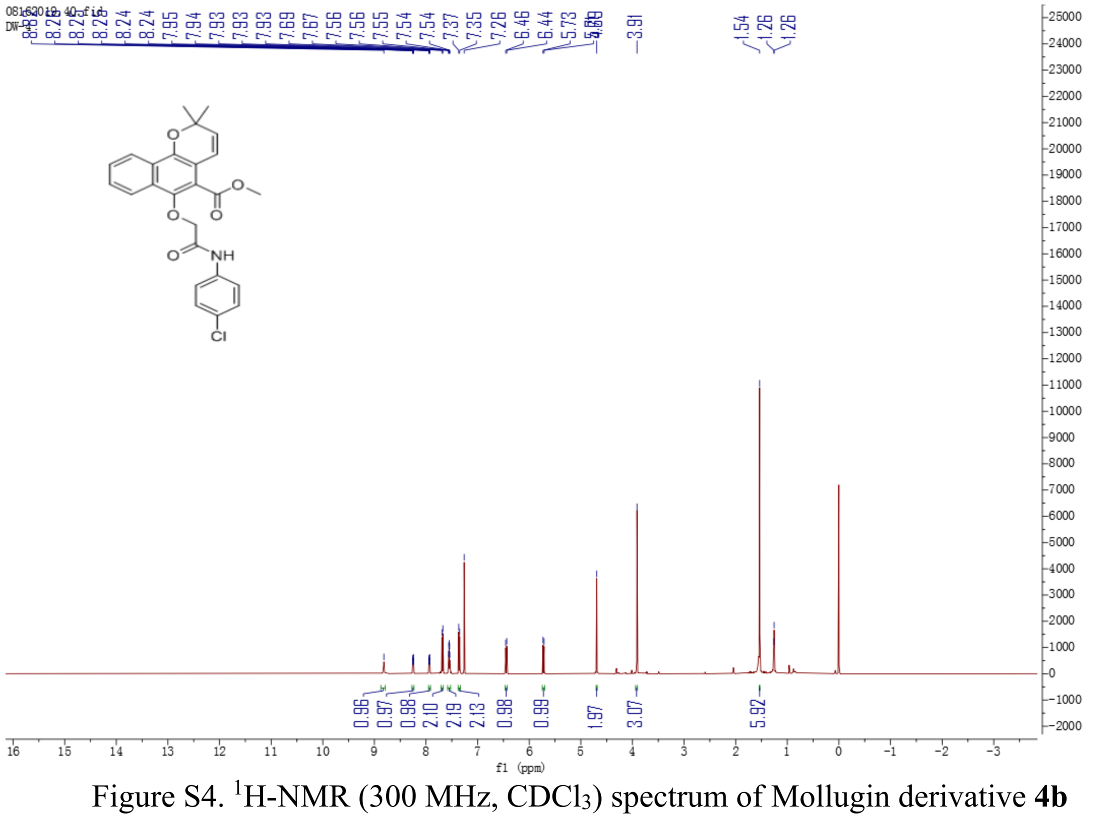

Figure S4. ^{1}H-NMR (300 MHz, $CDCl_3$) spectrum of Mollugin derivative **4b**

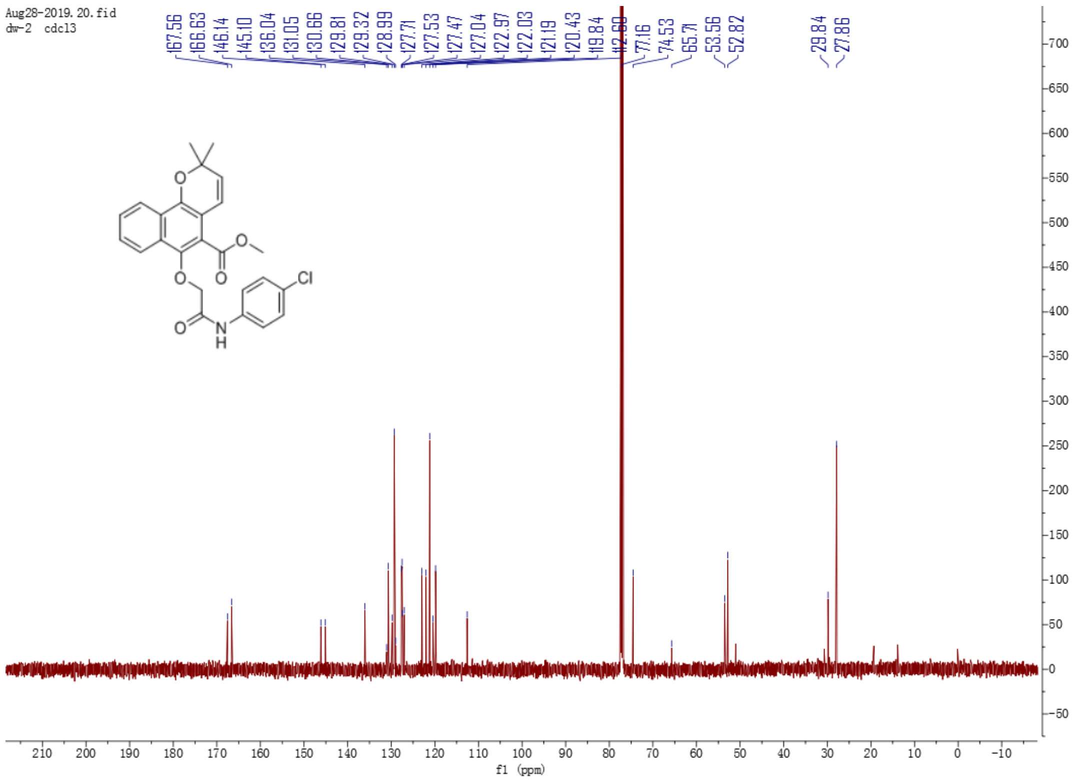

Figure S5. ^{13}C NMR (125 MHz, $CDCl_3$) spectrum of Mollugin derivative **4b**

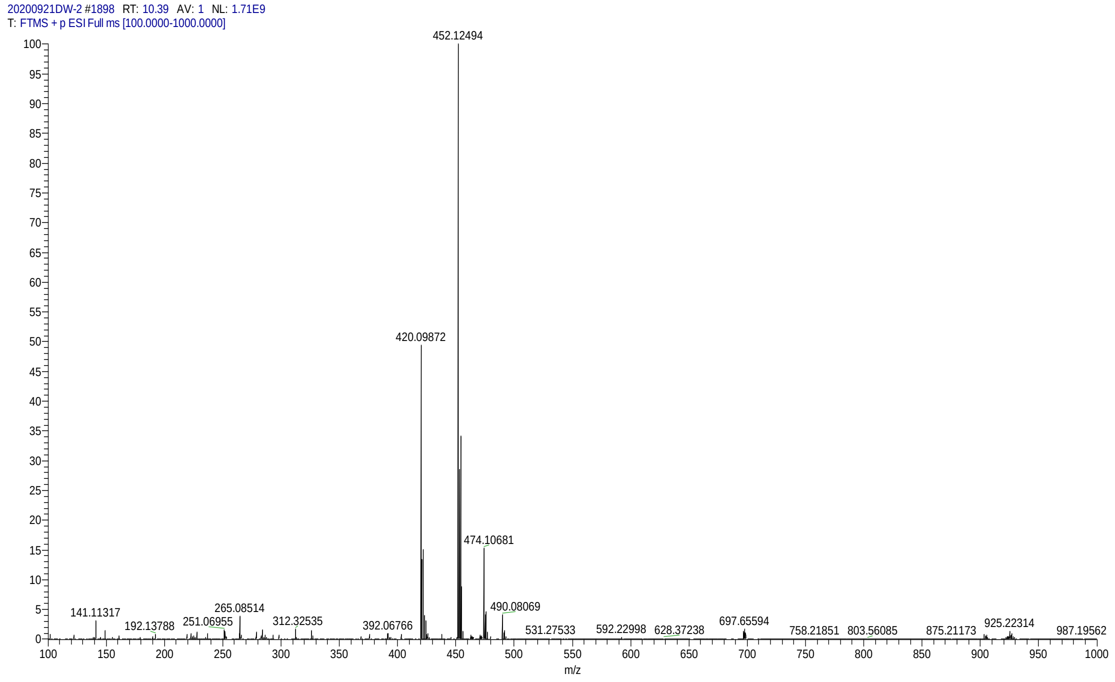

Figure S6. HRMS of Mollugin derivative **4b**

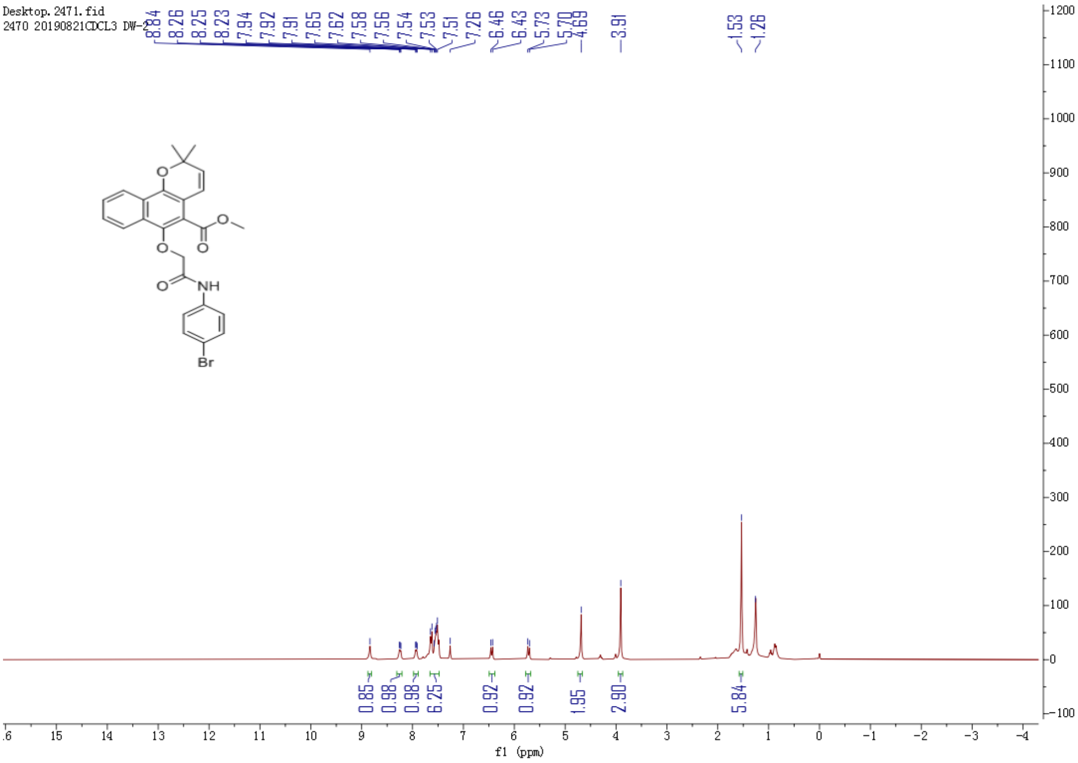

Figure S7. ^{1}H-NMR (300 MHz, $CDCl_3$) spectrum of Mollugin derivative **4c**

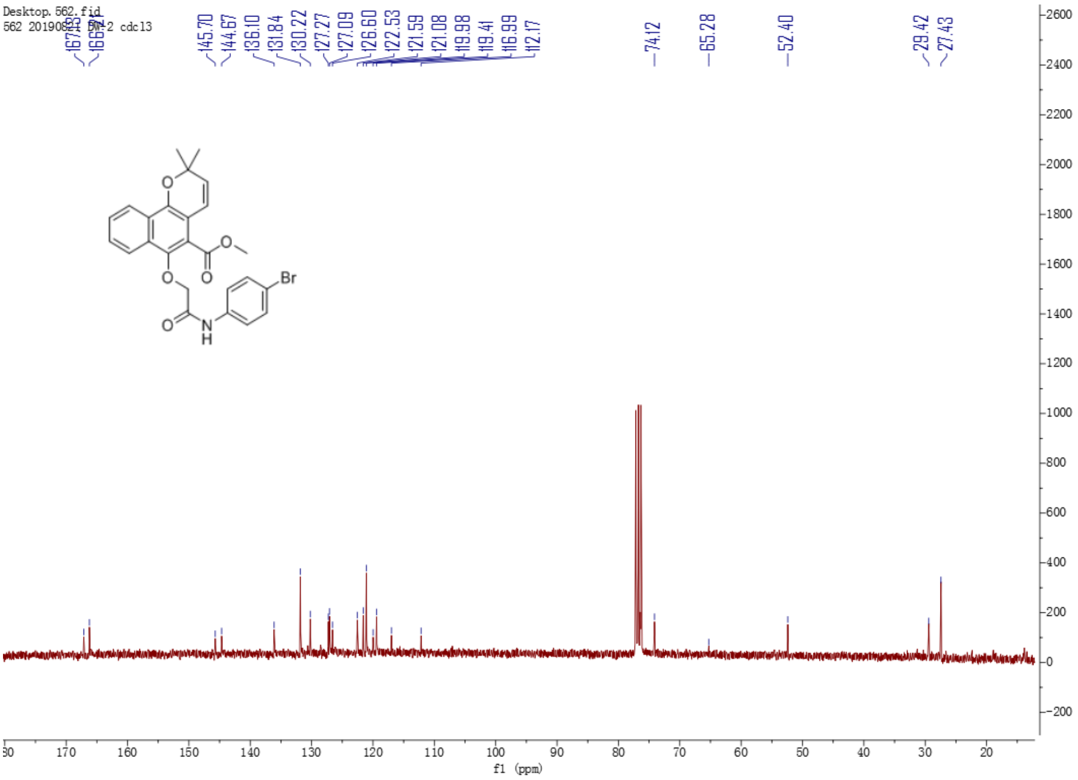

Figure S8. ^{13}C NMR (125 MHz, $CDCl_3$) spectrum of Mollugin derivative **4c**

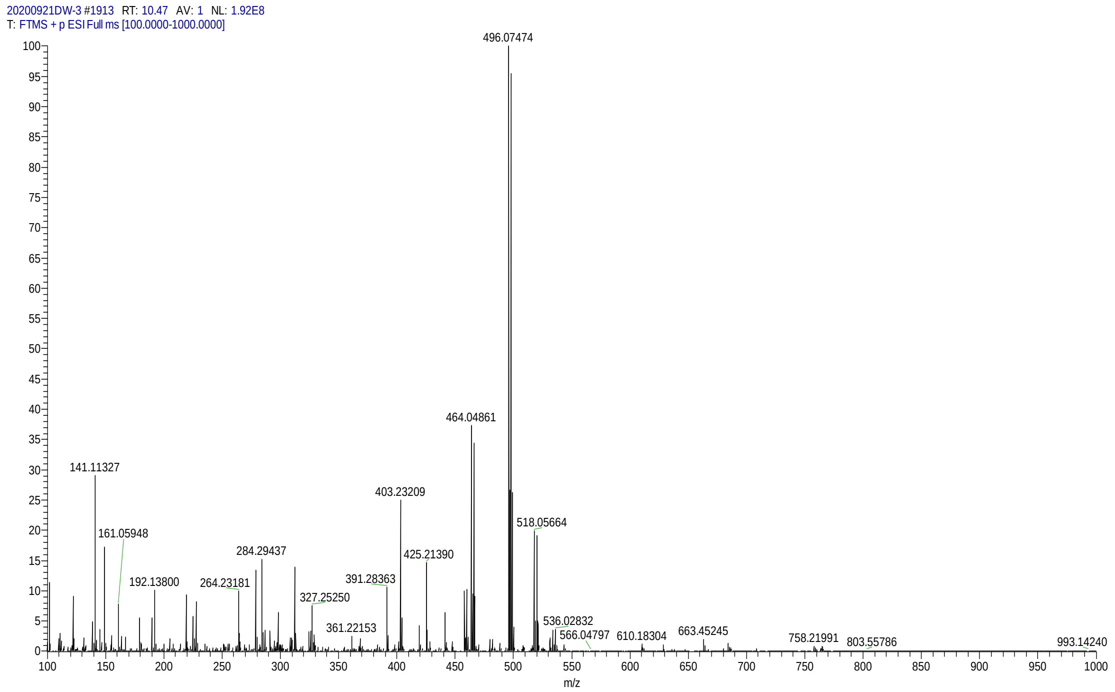

Figure S9. HRMS of Mollugin derivative **4c**

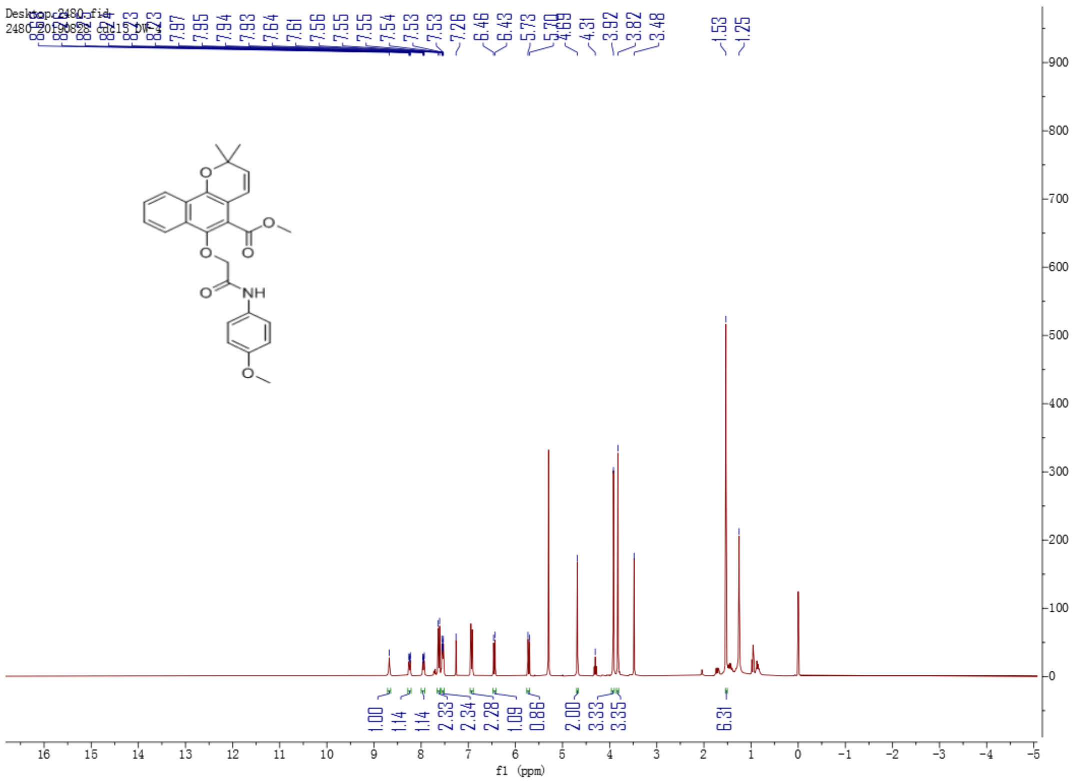

Figure S10. ^{1}H-NMR (300 MHz, $CDCl_3$) spectrum of Mollugin derivative **4d**

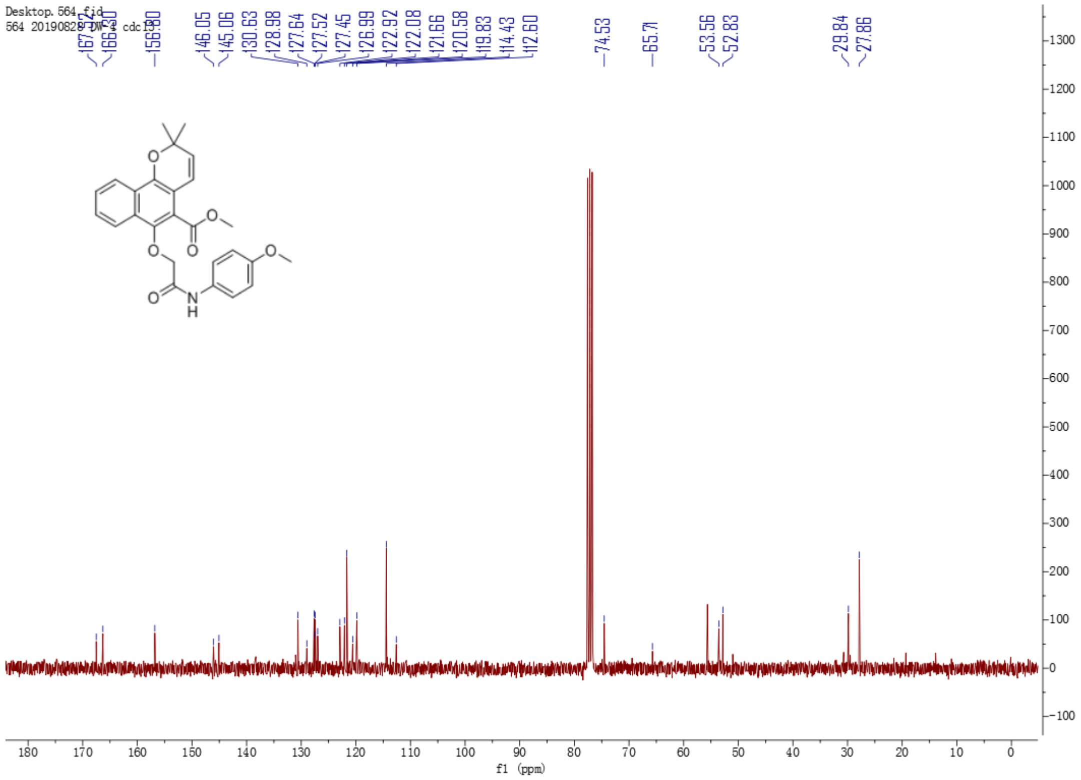

Figure S11. ^{13}C NMR (125 MHz, $CDCl_3$) spectrum of Mollugin derivative **4d**

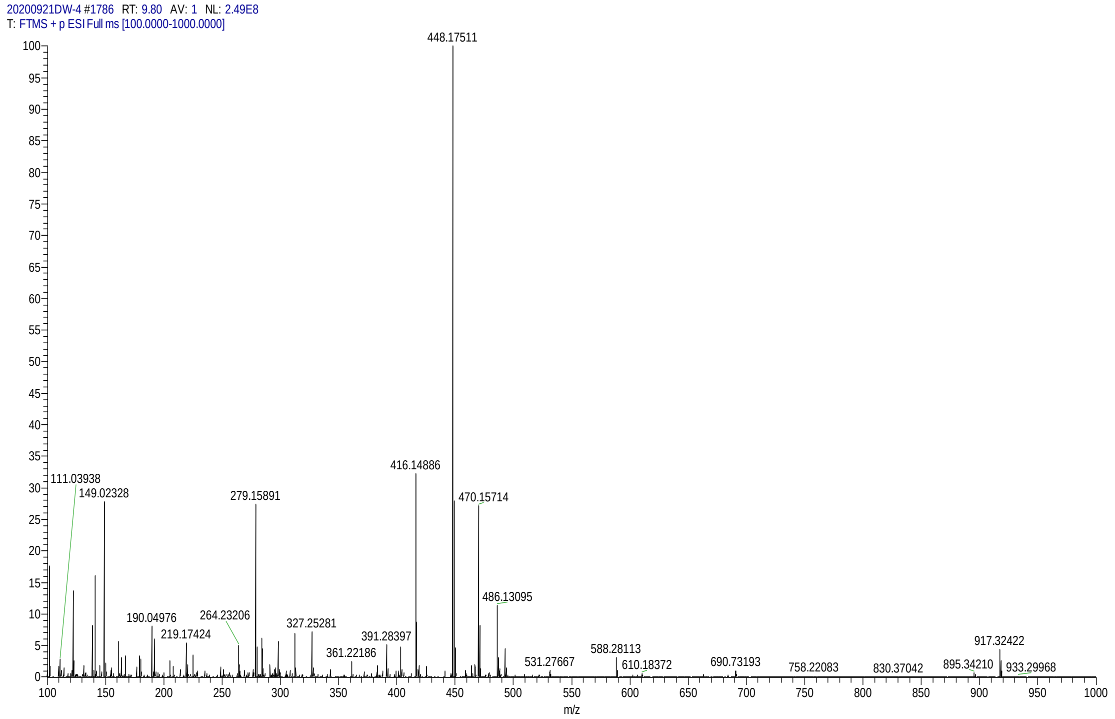

Figure S12. HRMS of Mollugin derivative **4d**

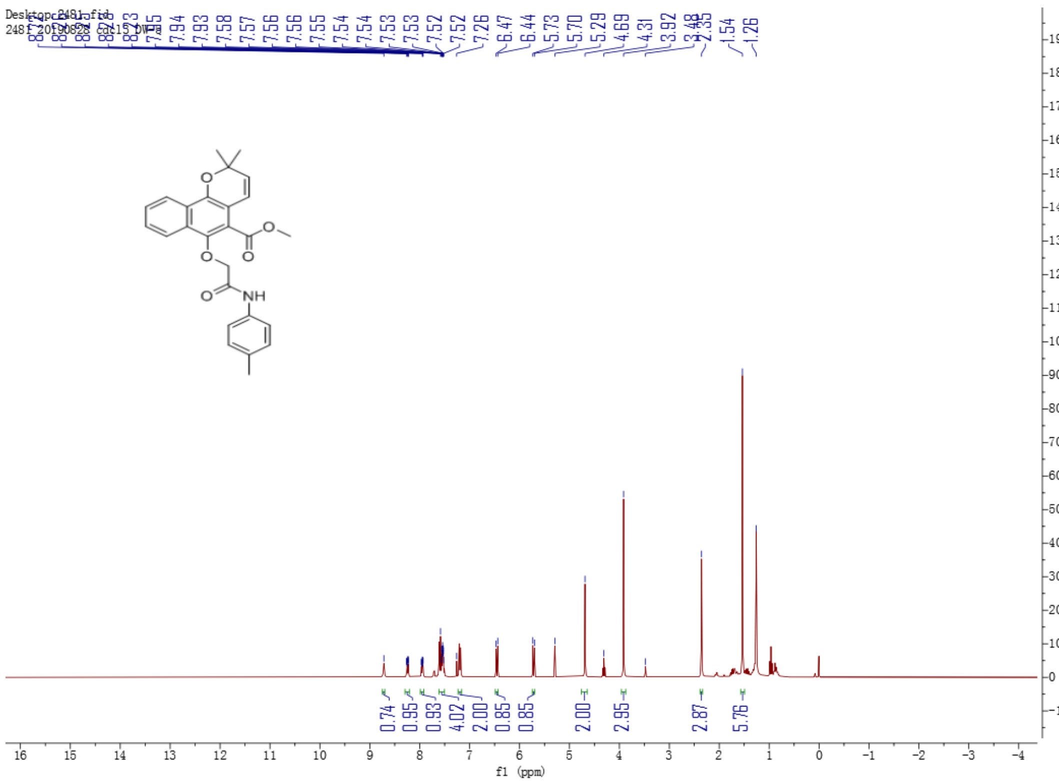

Figure S13. ^{1}H-NMR (300 MHz, $CDCl_3$) spectrum of Mollugin derivative **4e**

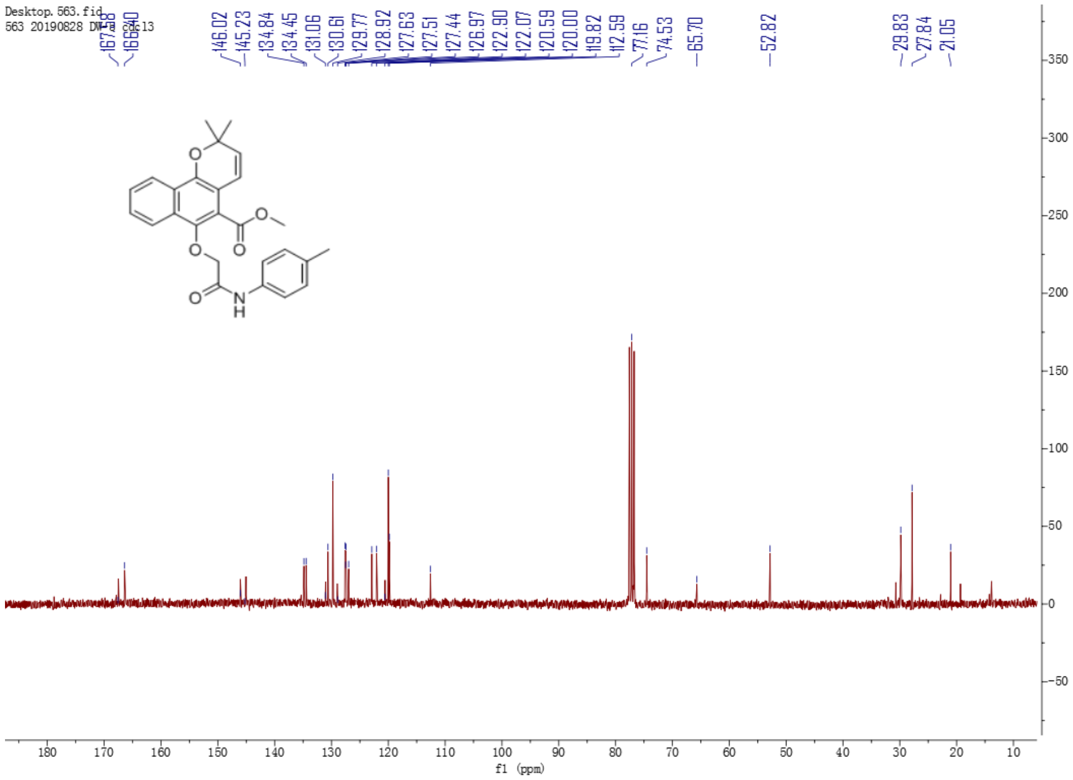

Figure S14. ^{13}C NMR (125 MHz, $CDCl_3$) spectrum of Mollugin derivative **4e**

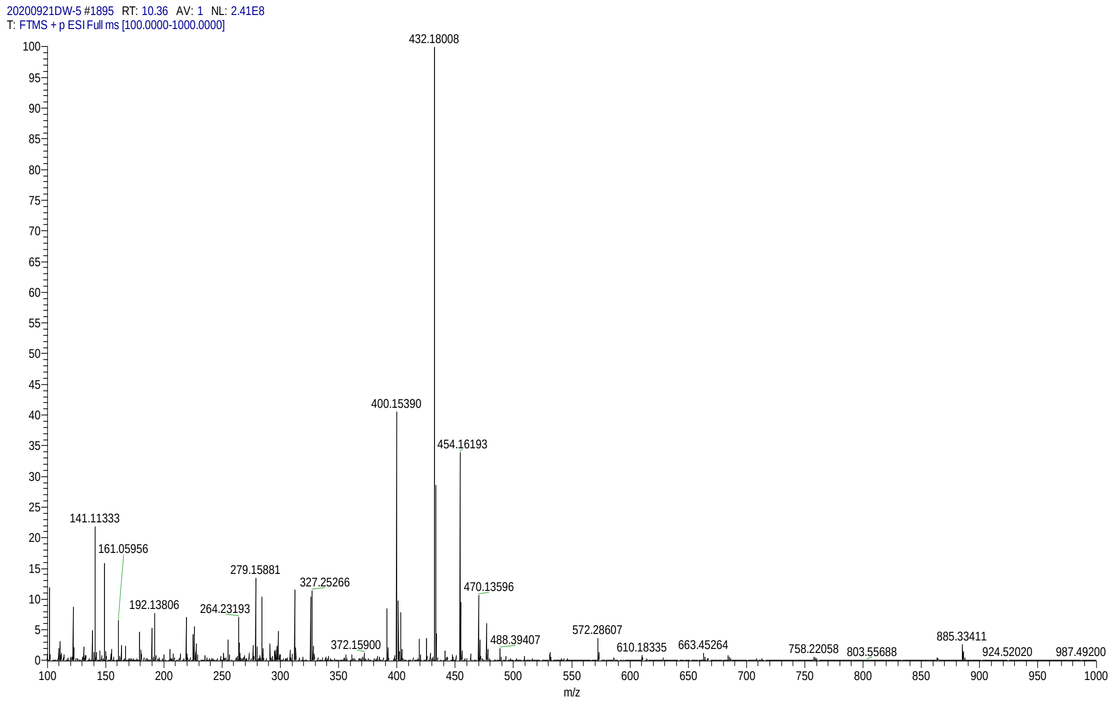

Figure S15. HRMS of Mollugin derivative **4e**

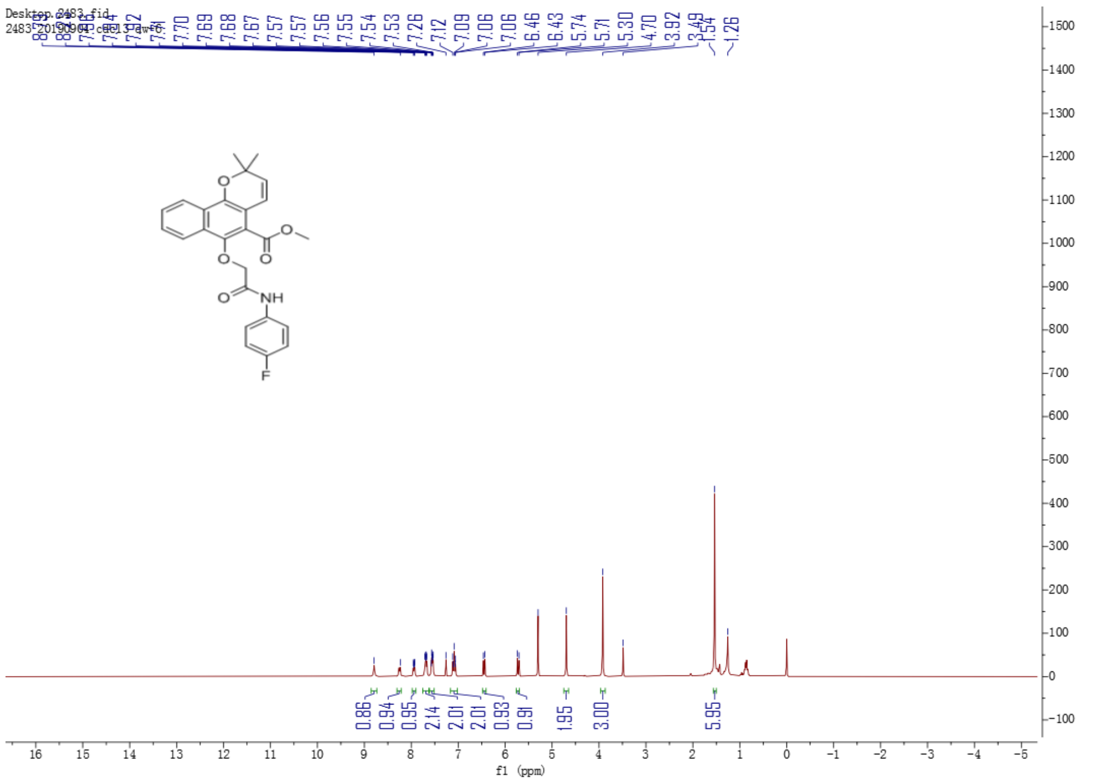

Figure S16. ^{1}H-NMR (300 MHz, $CDCl_3$) spectrum of Mollugin derivative **4f**

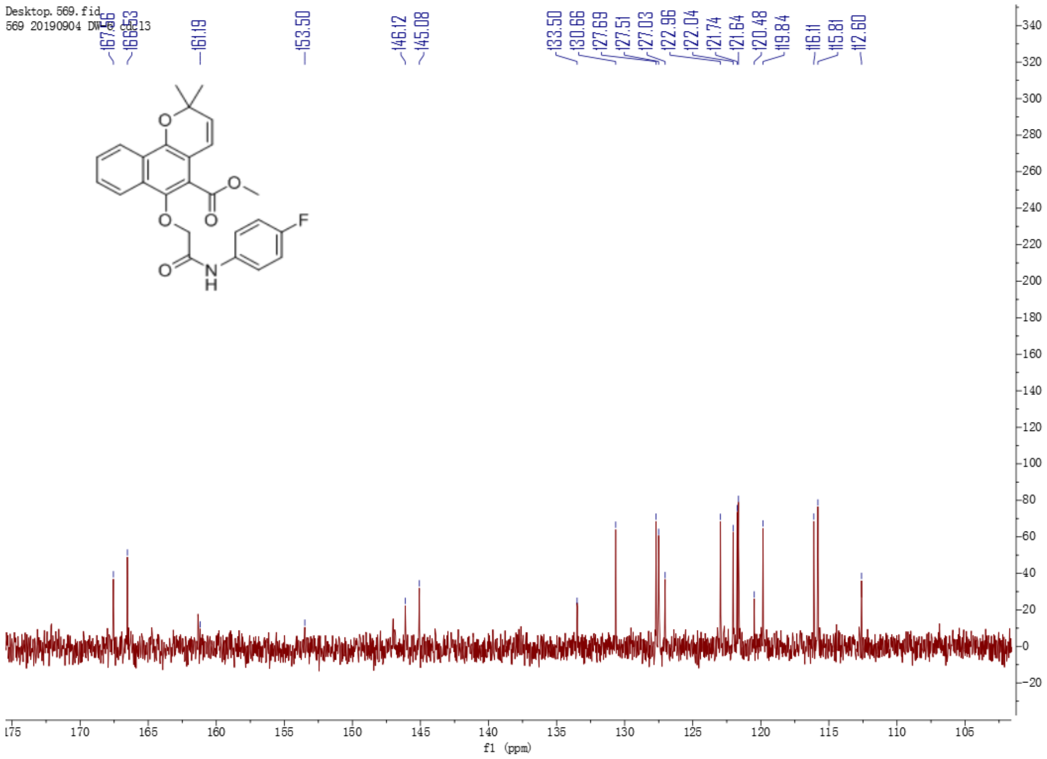

Figure S17. ^{13}C NMR (125 MHz, $CDCl_3$) spectrum of Mollugin derivative **4f**

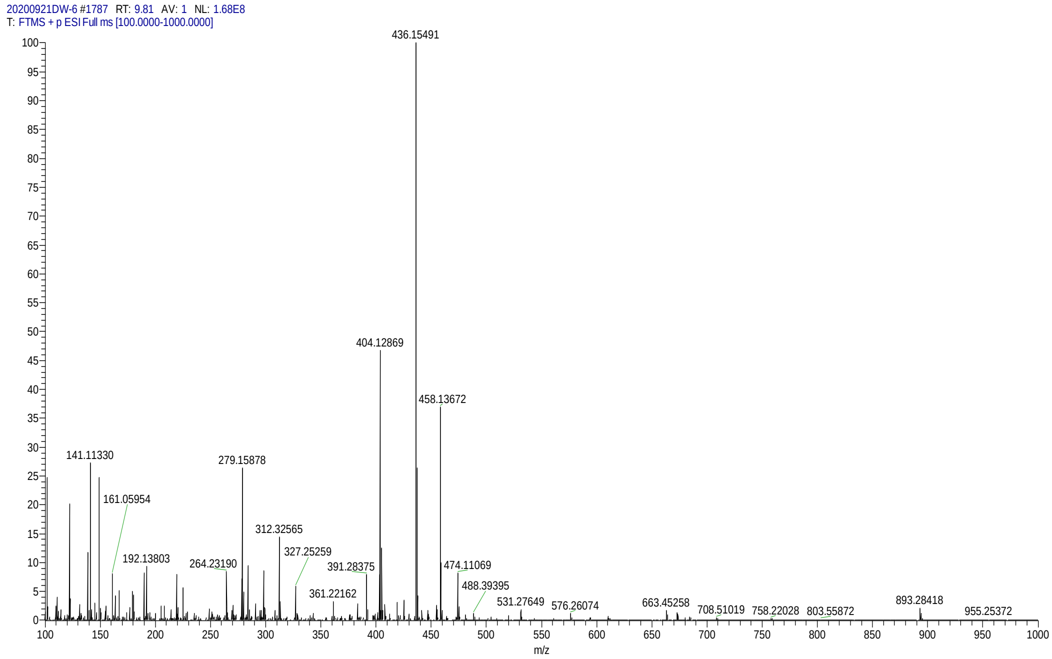

Figure S18. HRMS of Mollugin derivative **4f**

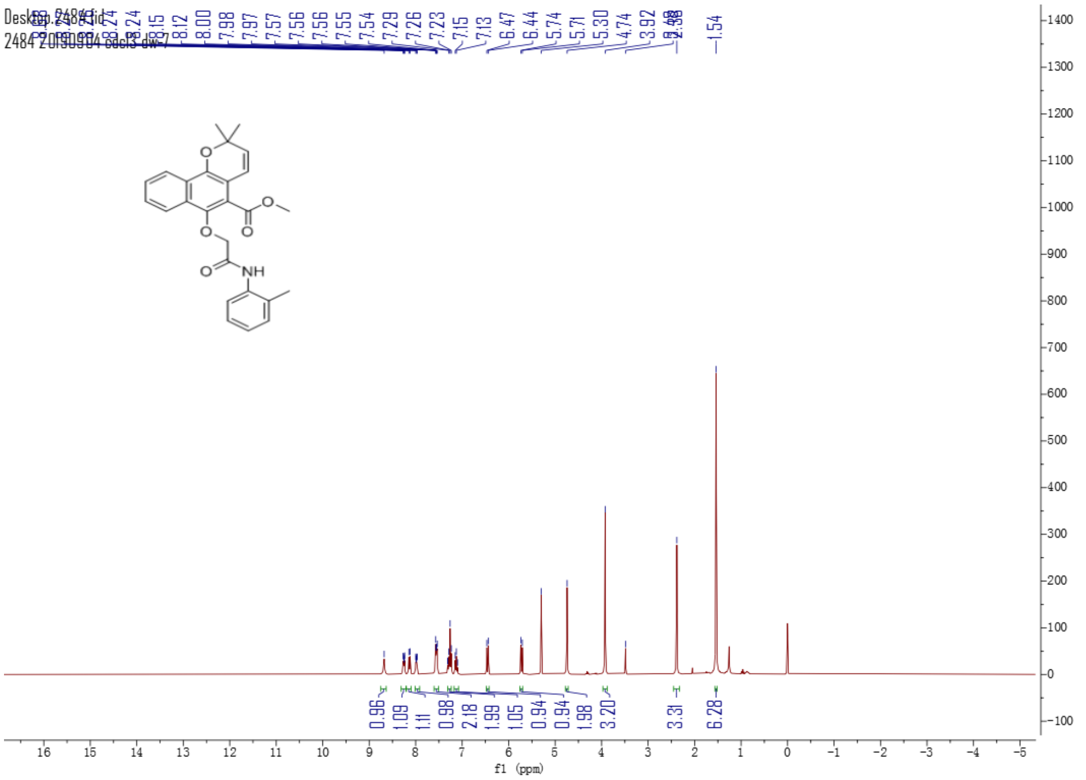

Figure S19. ^{1}H-NMR (300 MHz, $CDCl_3$) spectrum of Mollugin derivative **4g**

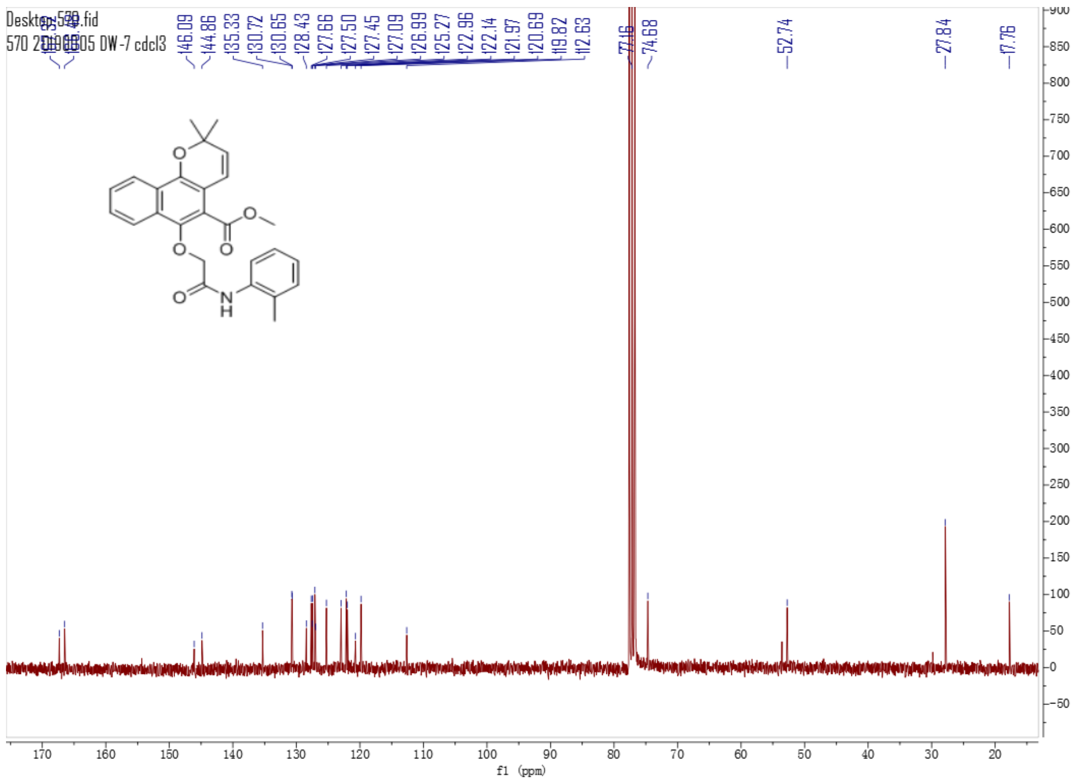

Figure S20. ^{13}C NMR (125 MHz, $CDCl_3$) spectrum of Mollugin derivative **4g**

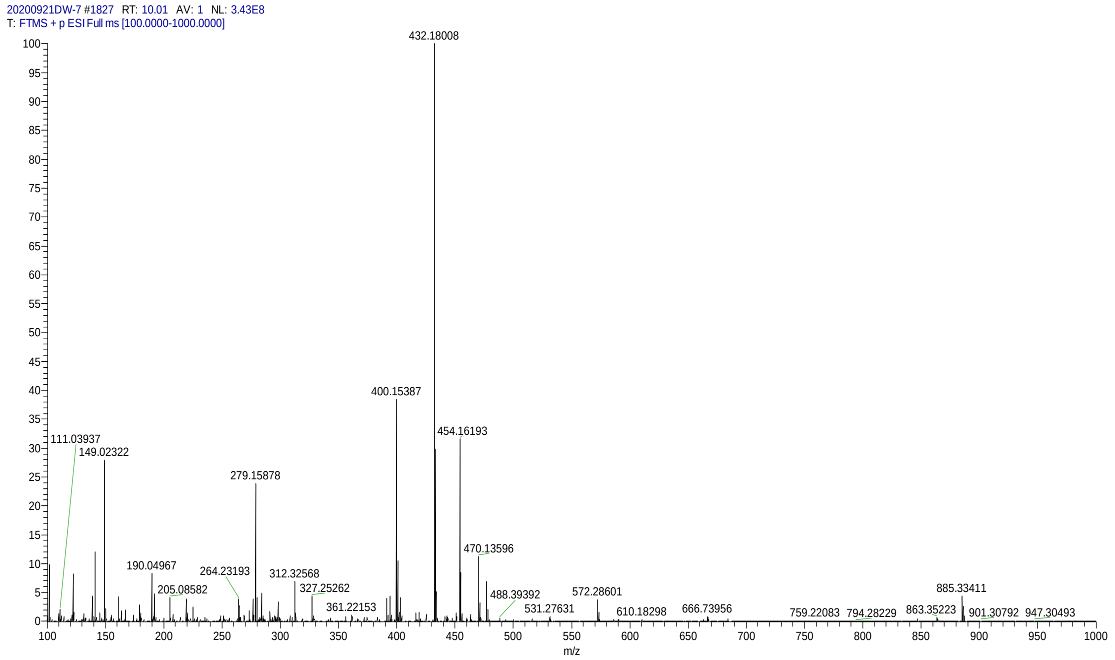

Figure S21. HRMS of Mollugin derivative **4g**

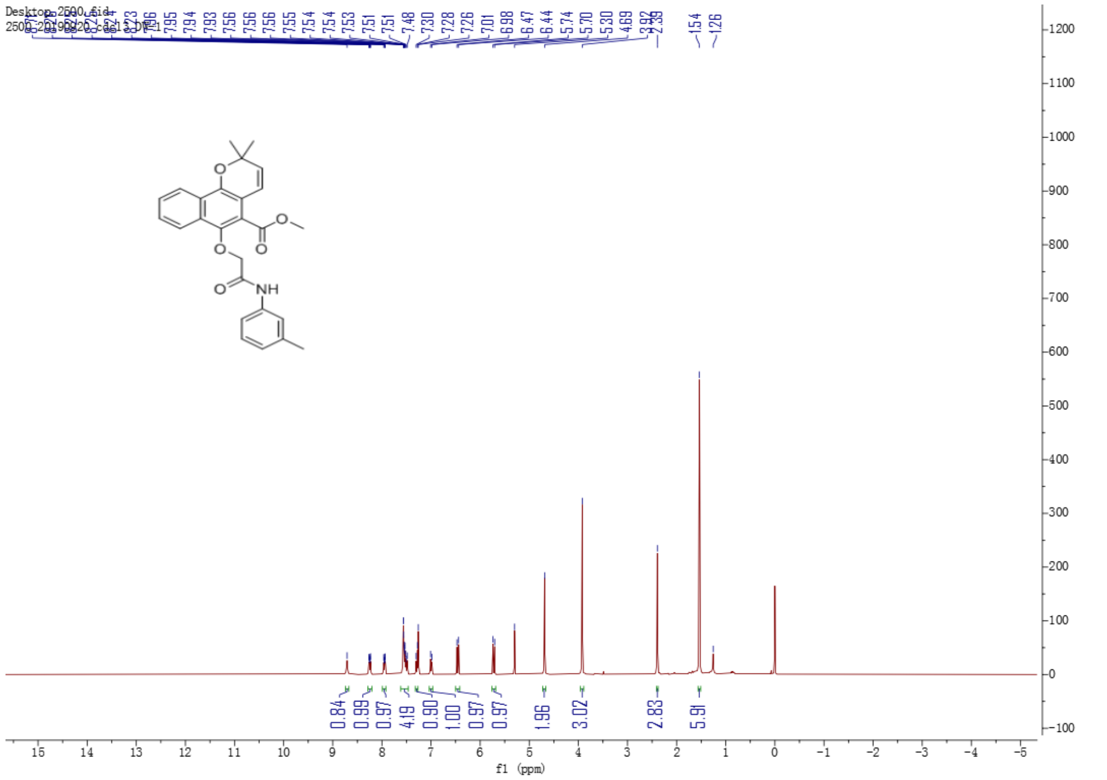

Figure S22. ^{1}H-NMR (300 MHz, $CDCl_3$) spectrum of Mollugin derivative **4h**

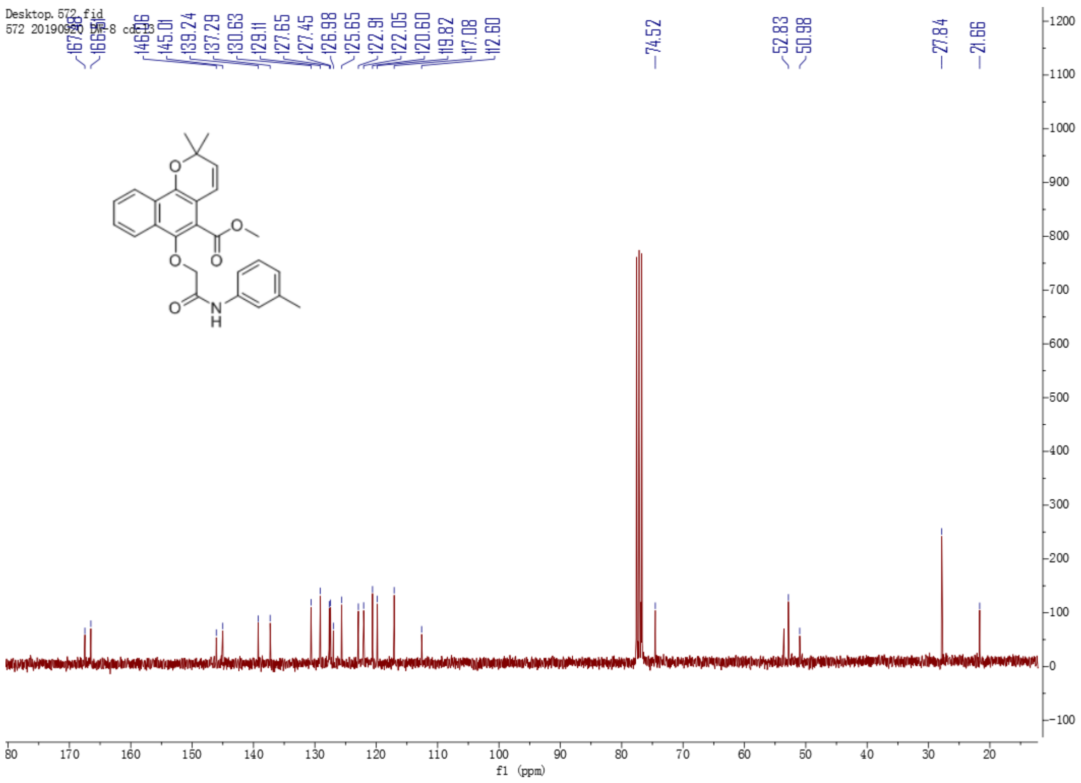

Figure S23. ^{13}C NMR (125 MHz, $CDCl_3$) spectrum of Mollugin derivative **4h**

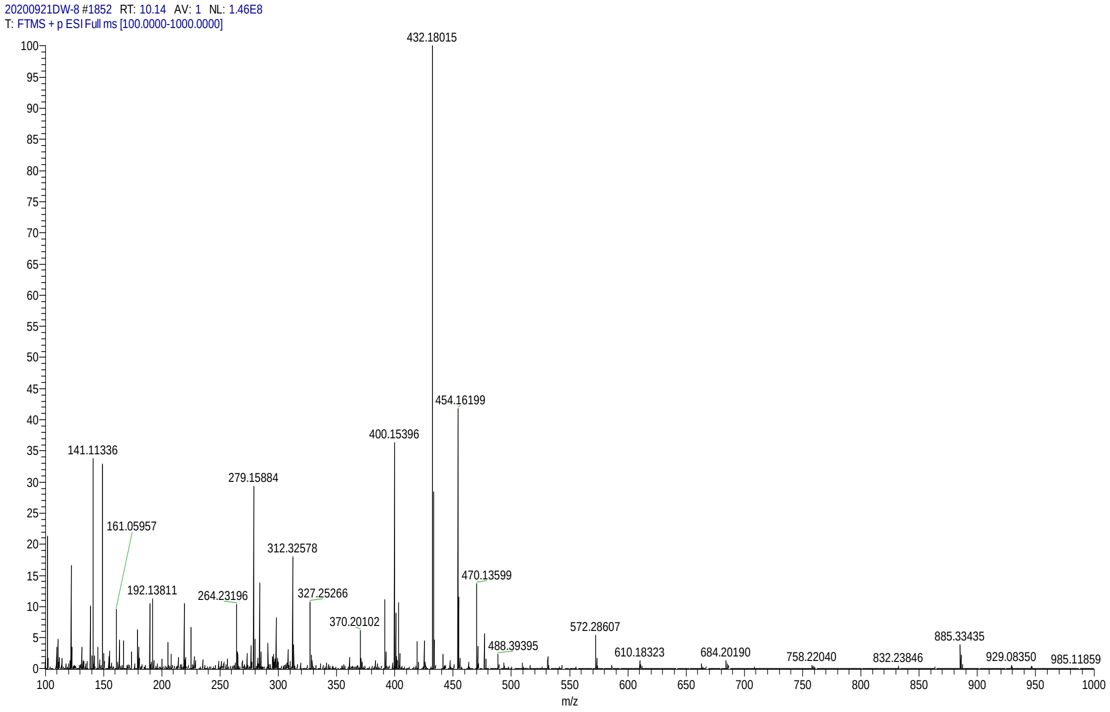

Figure S24. HRMS of Mollugin derivative **4h**

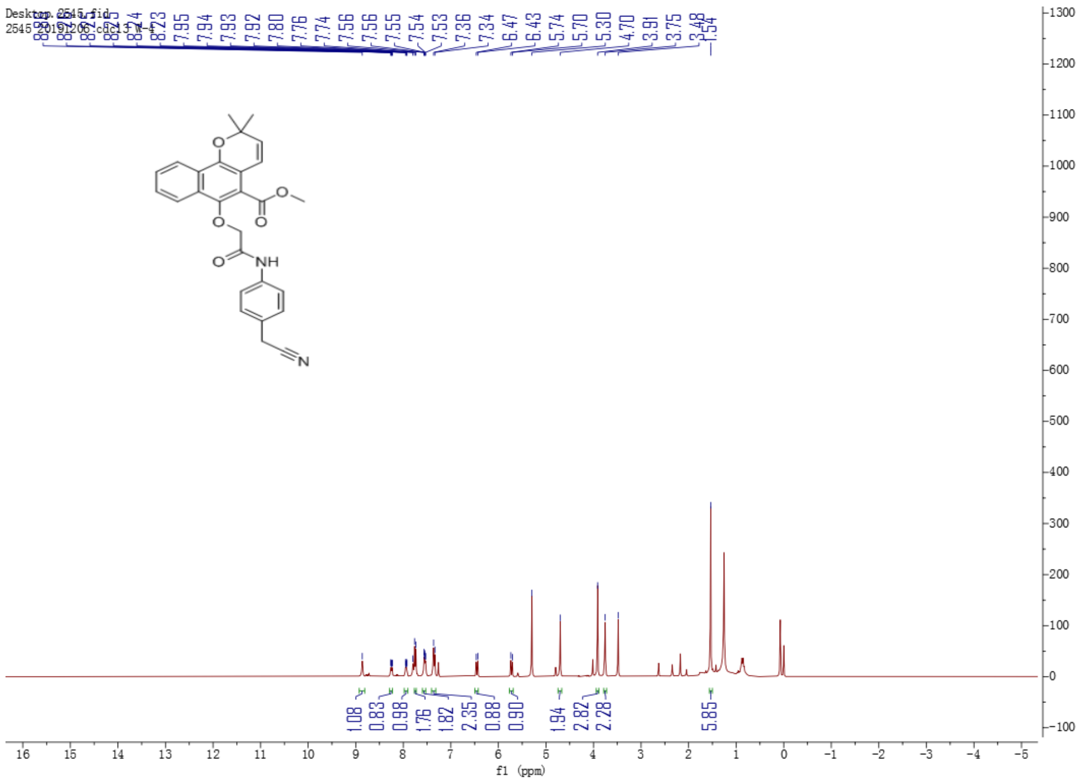

Figure S25. ^{1}H-NMR (300 MHz, $CDCl_3$) spectrum of Mollugin derivative **4i**

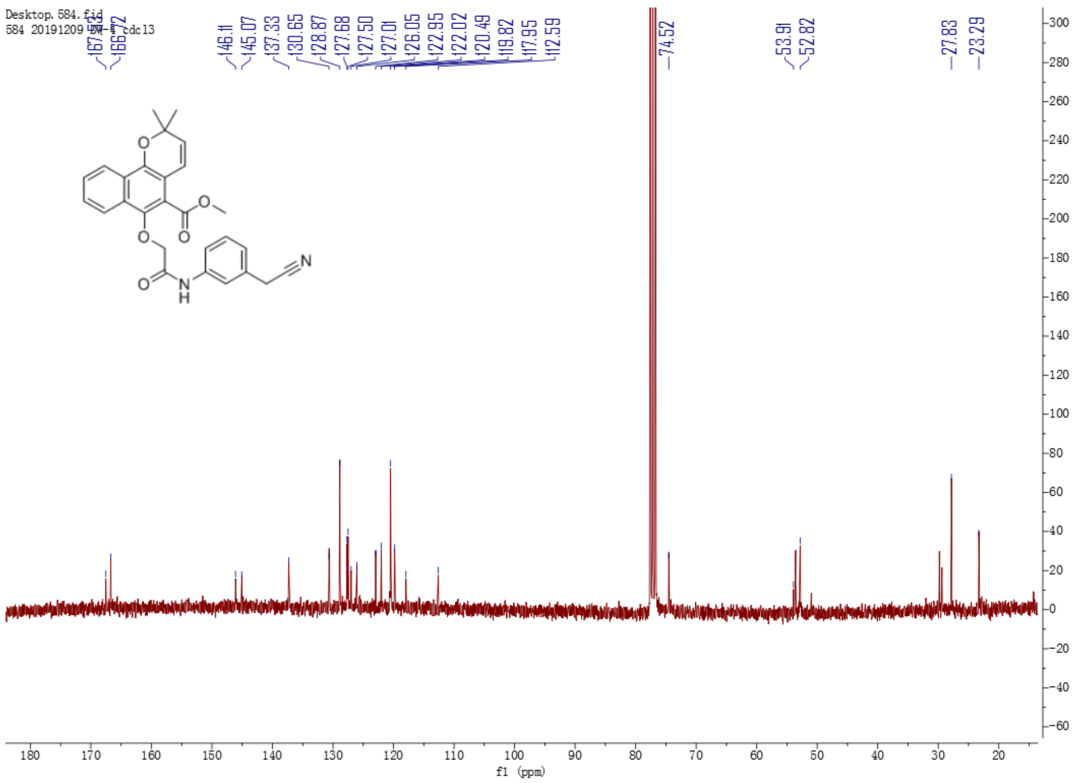

Figure S26. ^{13}C NMR (125 MHz, $CDCl_3$) spectrum of Mollugin derivative **4i**

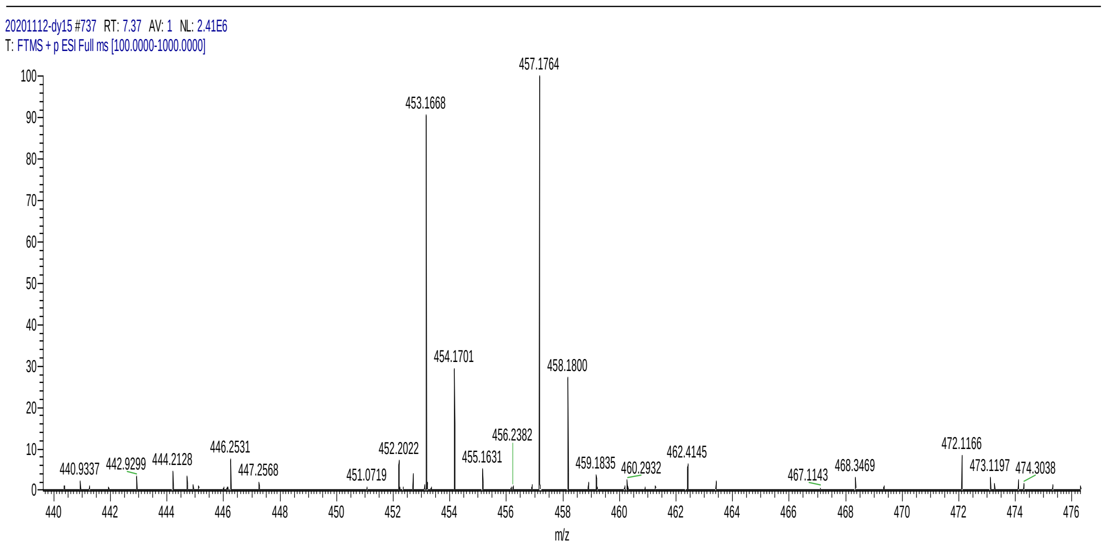

Figure S27. HRMS of Mollugin derivative **4i**

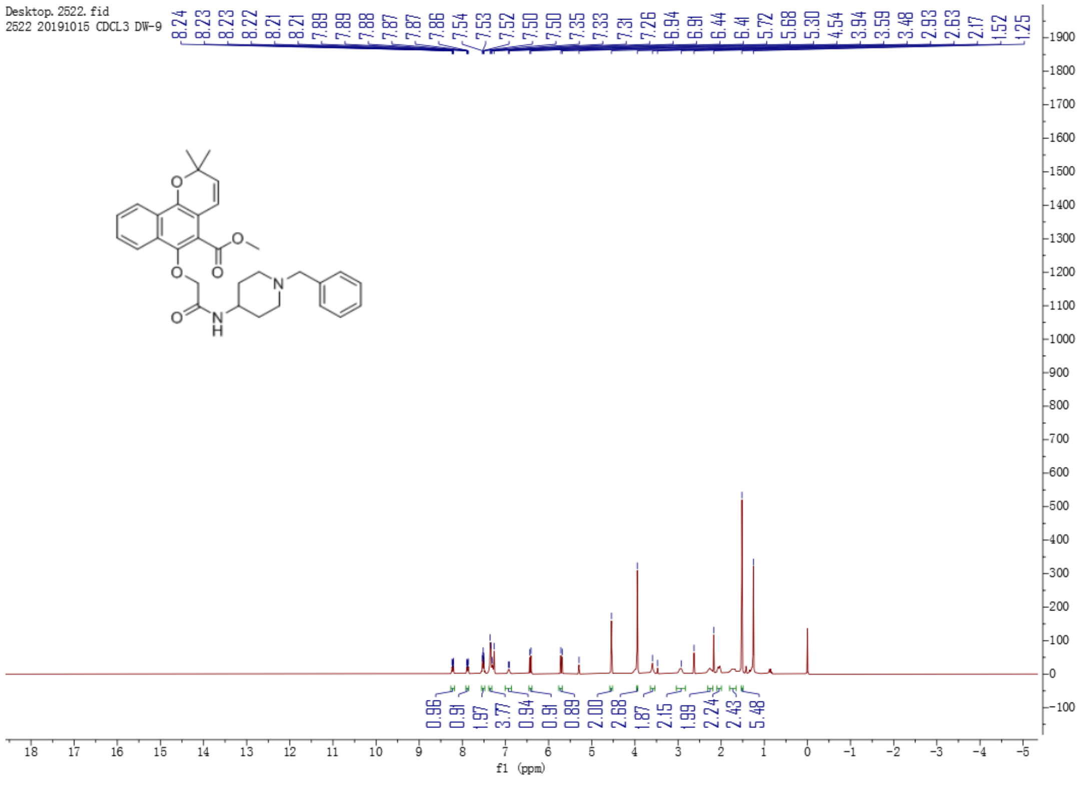

Figure S28. ^{1}H-NMR (300 MHz, $CDCl_3$) spectrum of Mollugin derivative **6a**

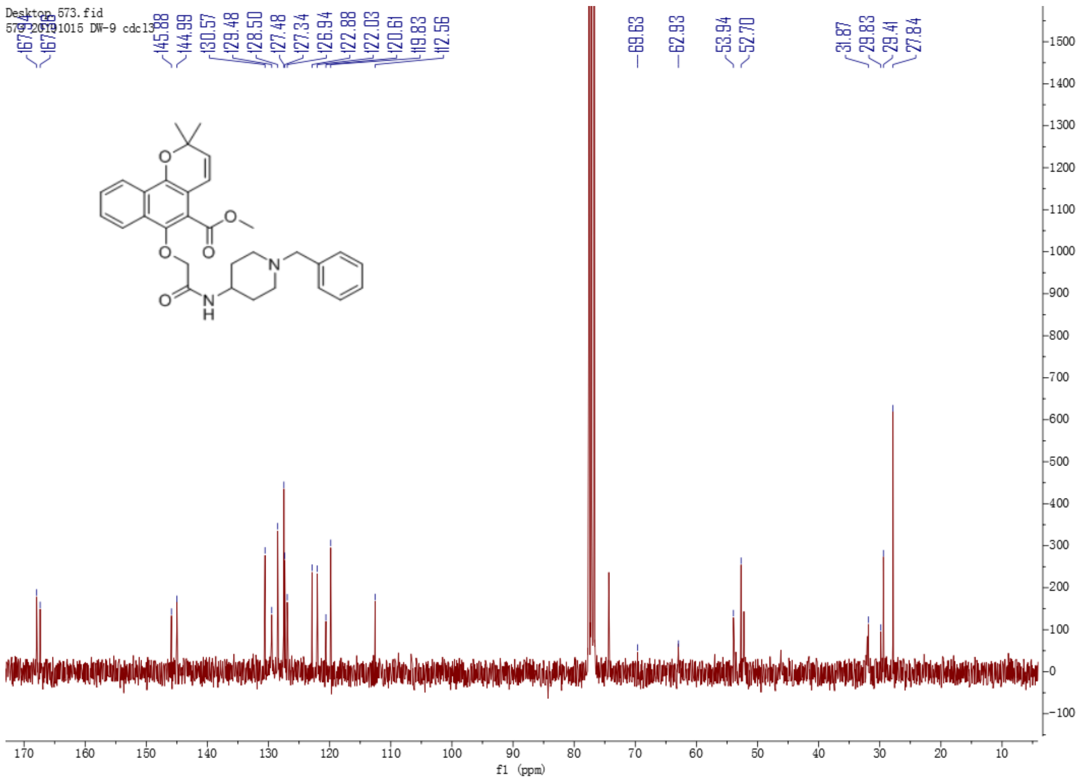

Figure S29. ^{13}C NMR (125 MHz, $CDCl_3$) spectrum of Mollugin derivative **6**[a]

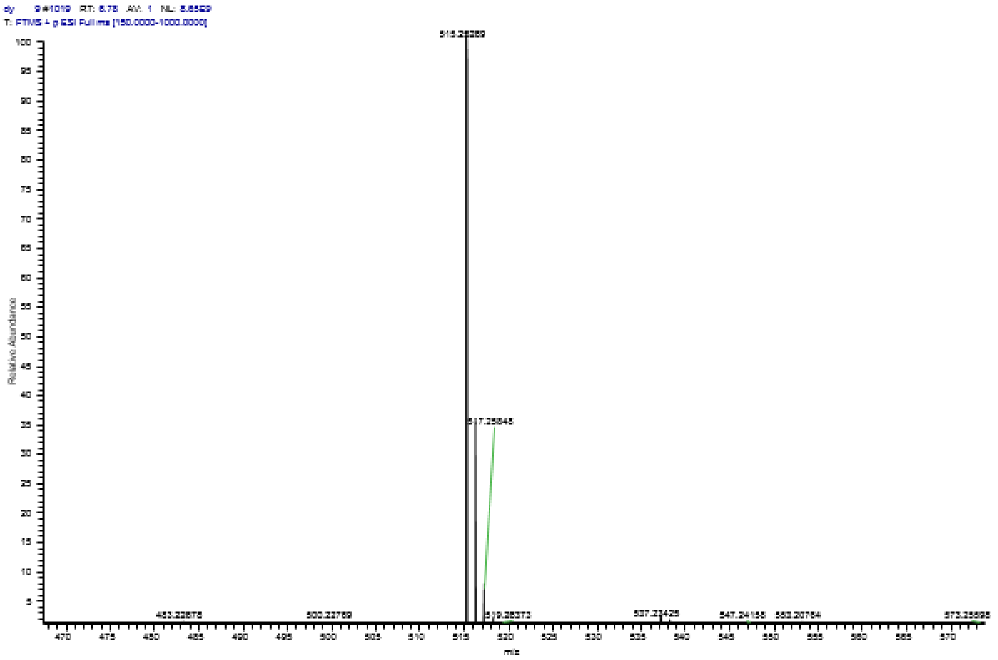

Figure S30. HRMS of Mollugin derivative **6a**

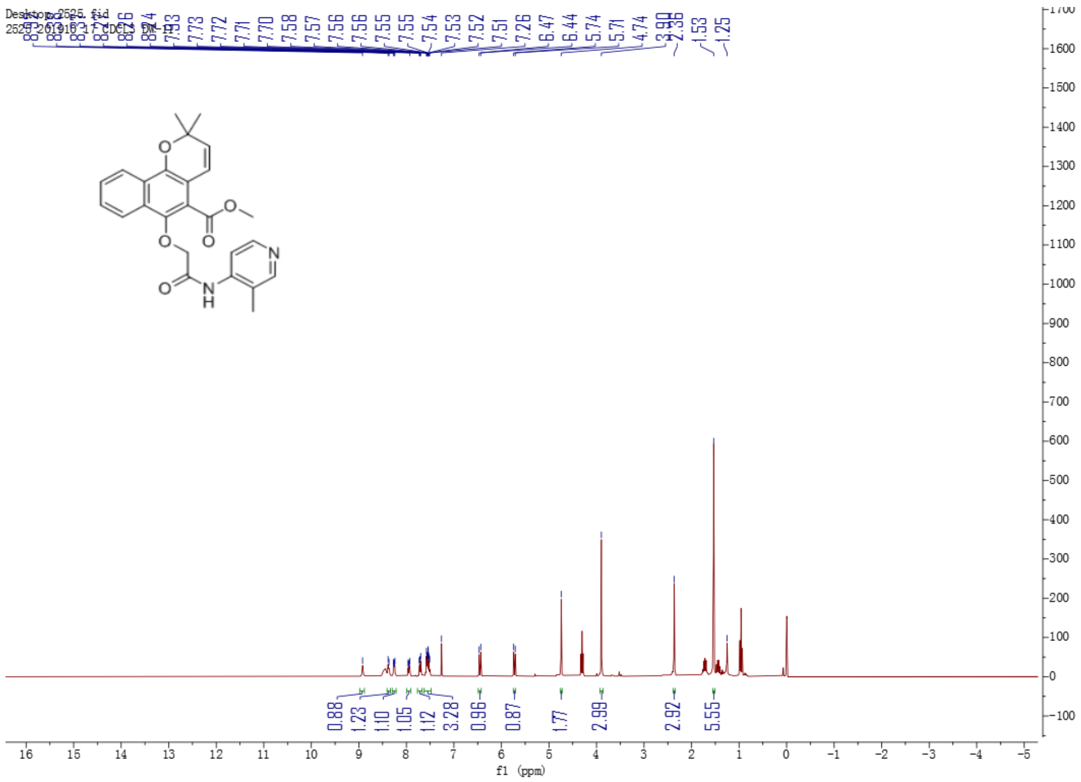

Figure S31. ^{1}H-NMR (300 MHz, $CDCl_3$) spectrum of Mollugin derivative **6b**

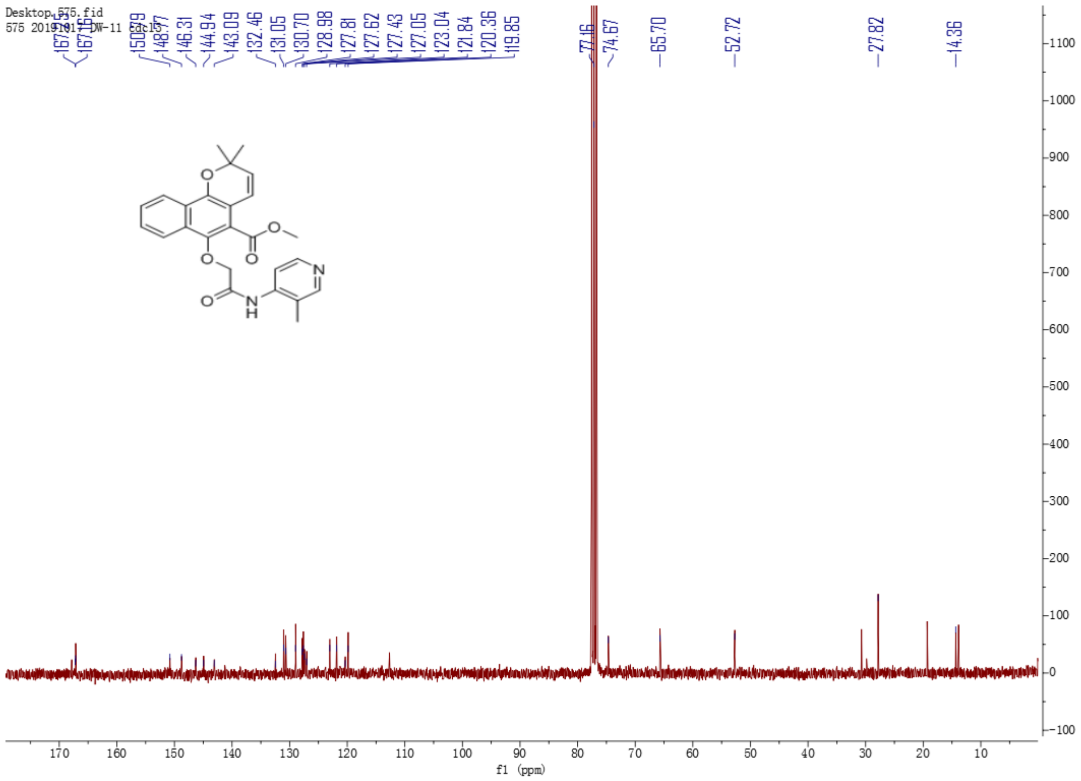

Figure S32. ^{13}C NMR (125 MHz, $CDCl_3$) spectrum of Mollugin derivative **6b**

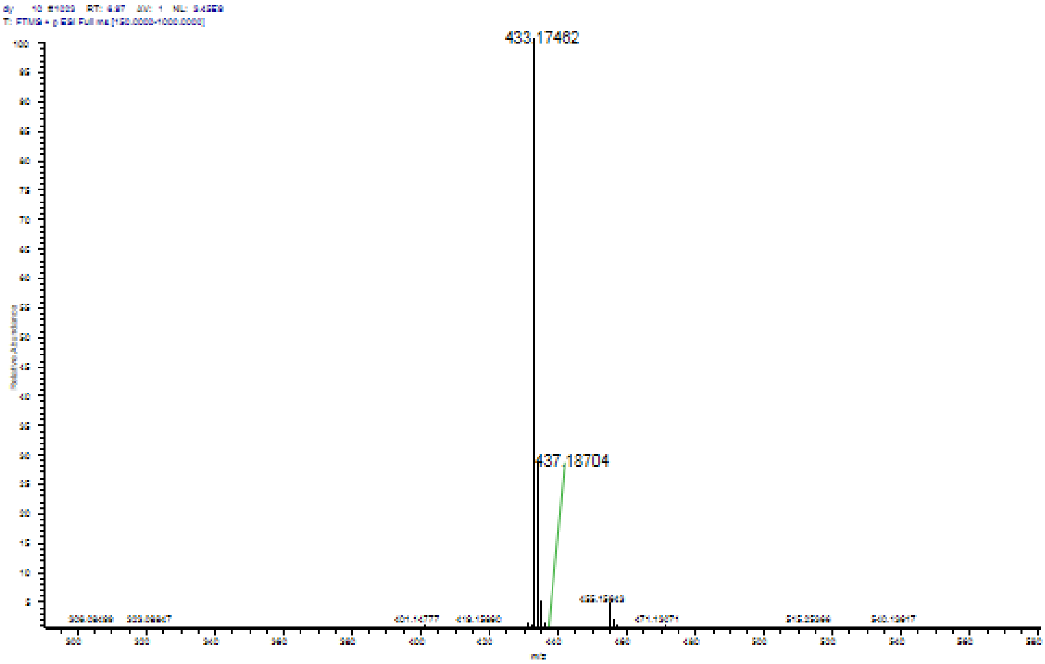

Figure S33. HRMS of Mollugin derivative **6b**

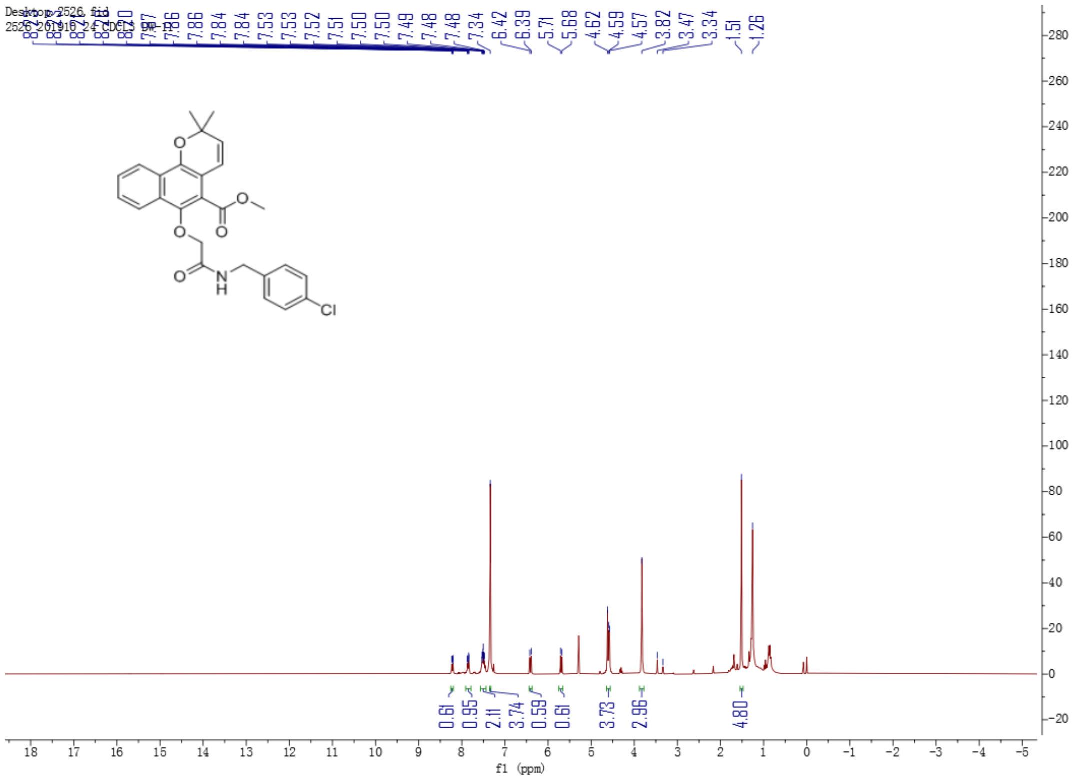

Figure S34. ^{1}H-NMR (300 MHz, $CDCl_3$) spectrum of Mollugin derivative **6c**

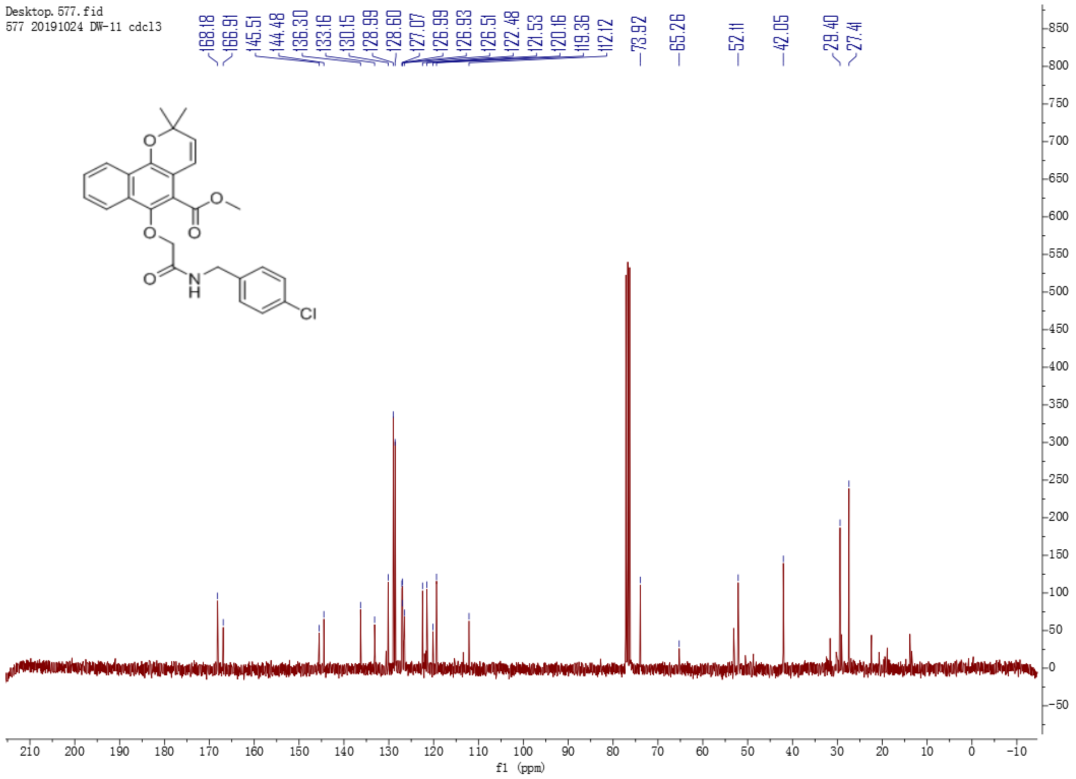

Figure S35. ^{13}C NMR (125 MHz, $CDCl_3$) spectrum of Mollugin derivative **6c**

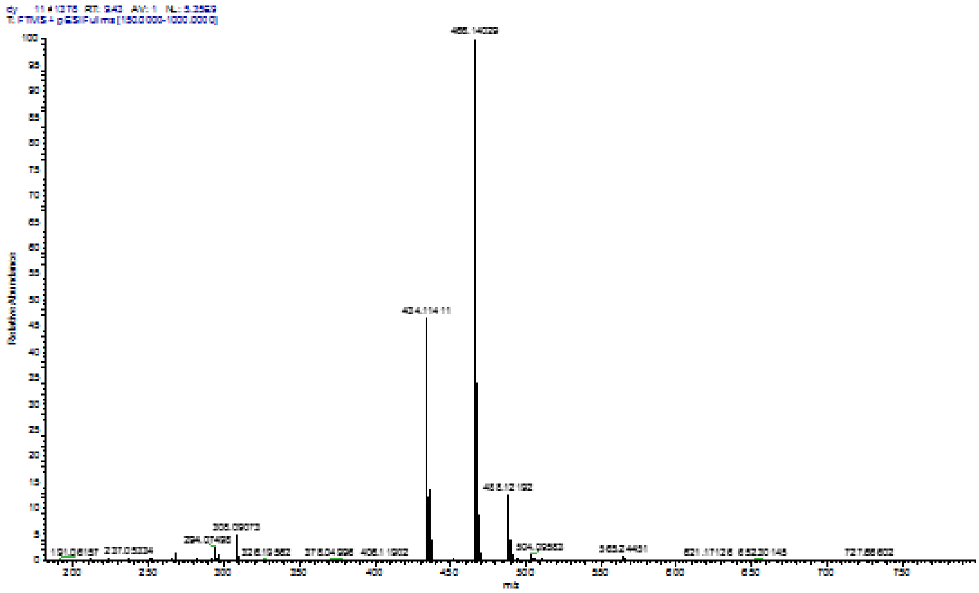

Figure S36. HRMS of Mollugin derivative **6c**

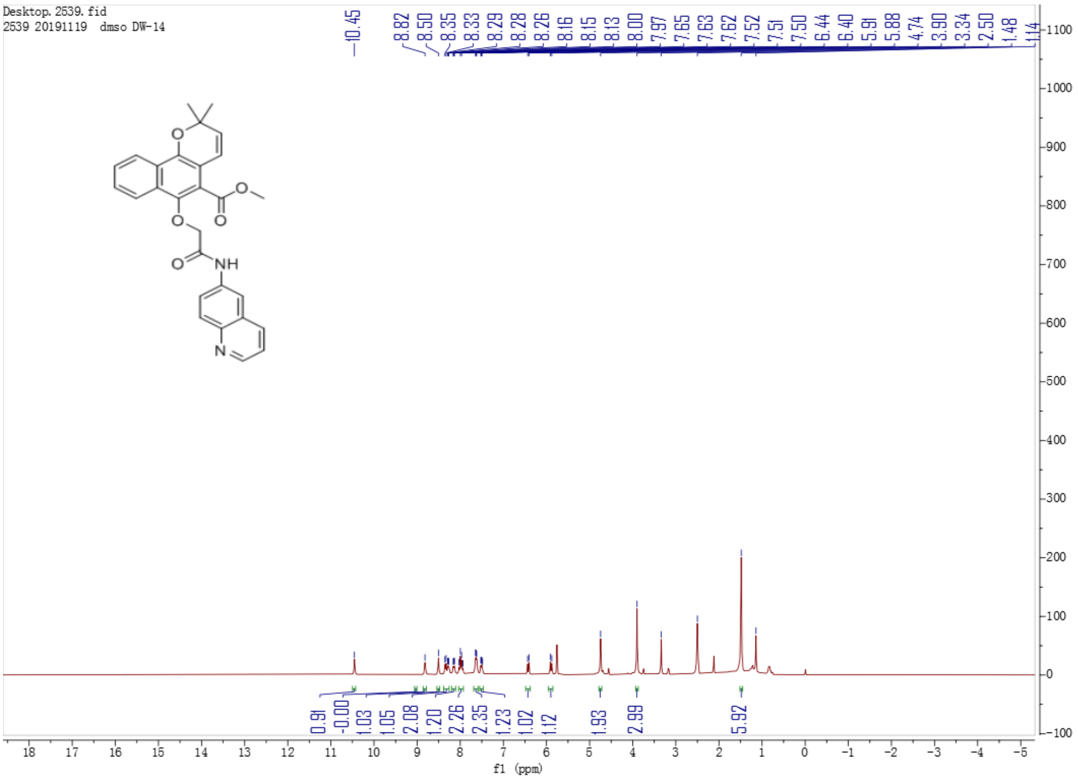

Figure S37. ^{1}H-NMR (300 MHz, DMSO-d_6) spectrum of Mollugin derivative **6d**

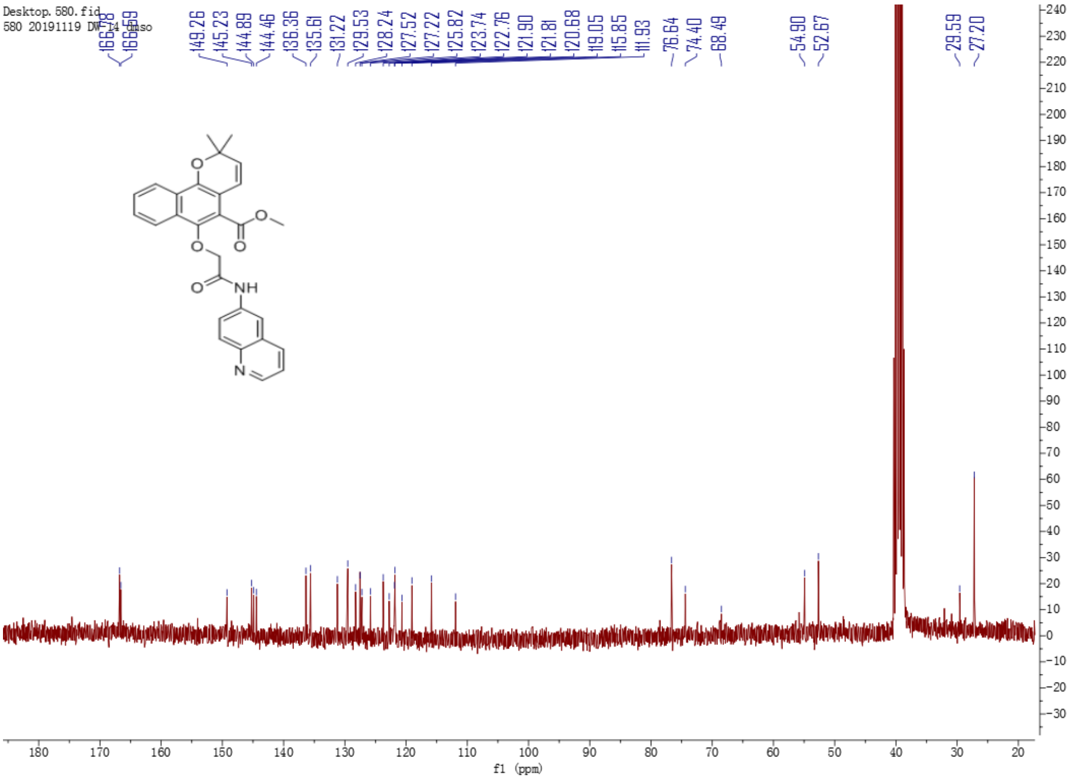

Figure S38. ^{13}C NMR (125 MHz, DMSO-d_6) spectrum of Mollugin derivative **6d**

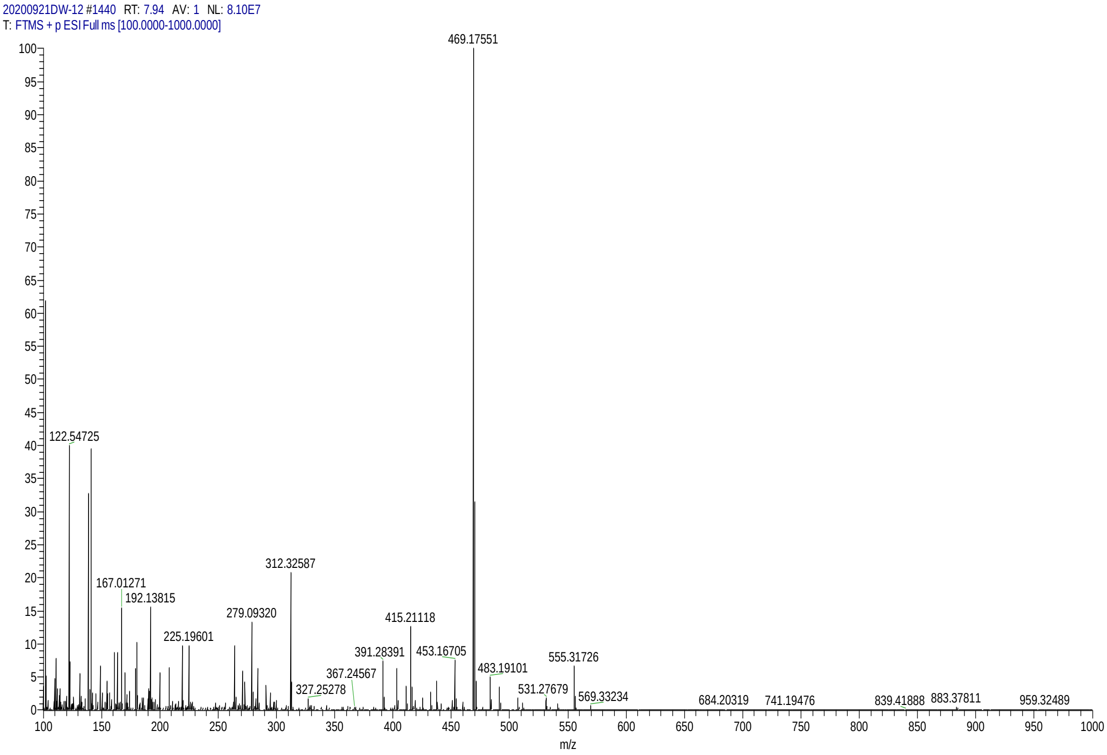

Figure S39. HRMS of Mollugin derivative **6d**

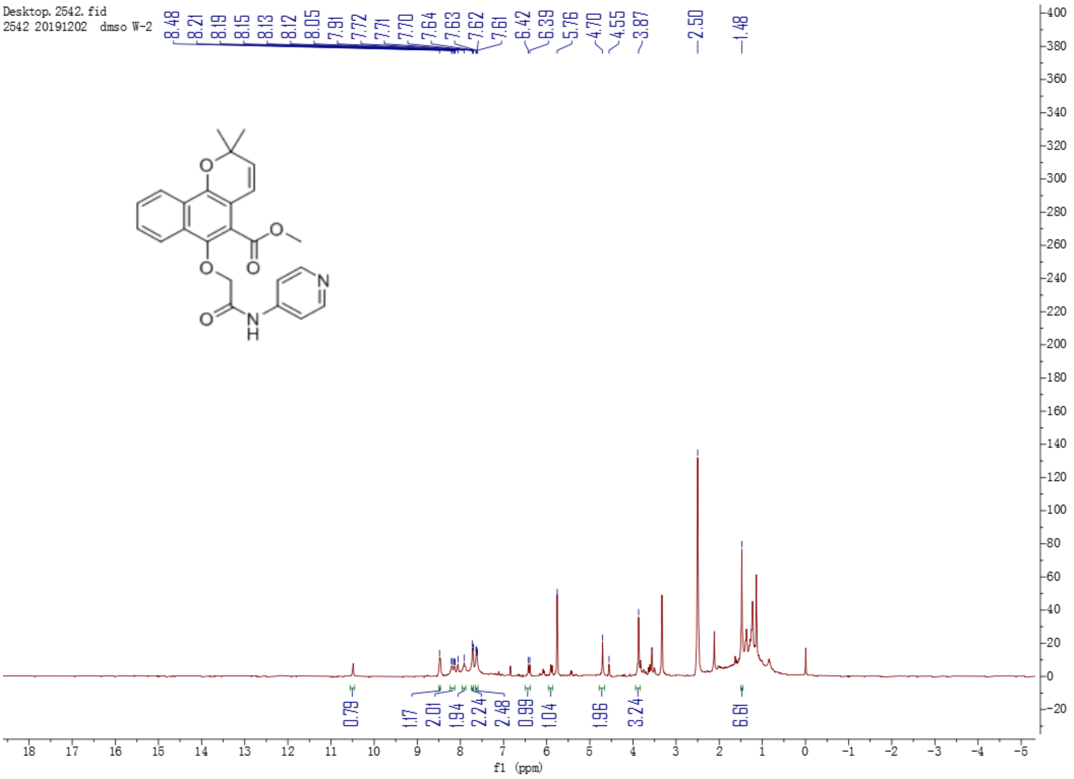

Figure S40. ^{1}H-NMR (300 MHz, DMSO-d_6) spectrum of Mollugin derivative **6e**

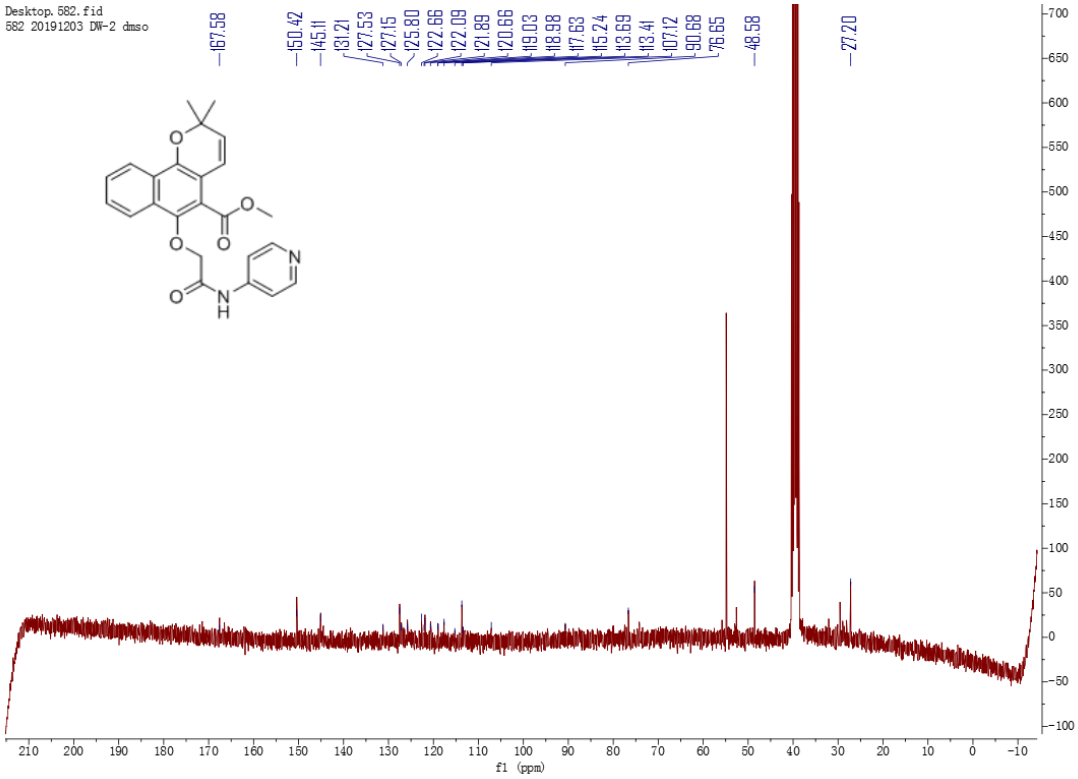

Figure S41. ^{13}C NMR (125 MHz, DMSO-d_6) spectrum of Mollugin derivative **6e**

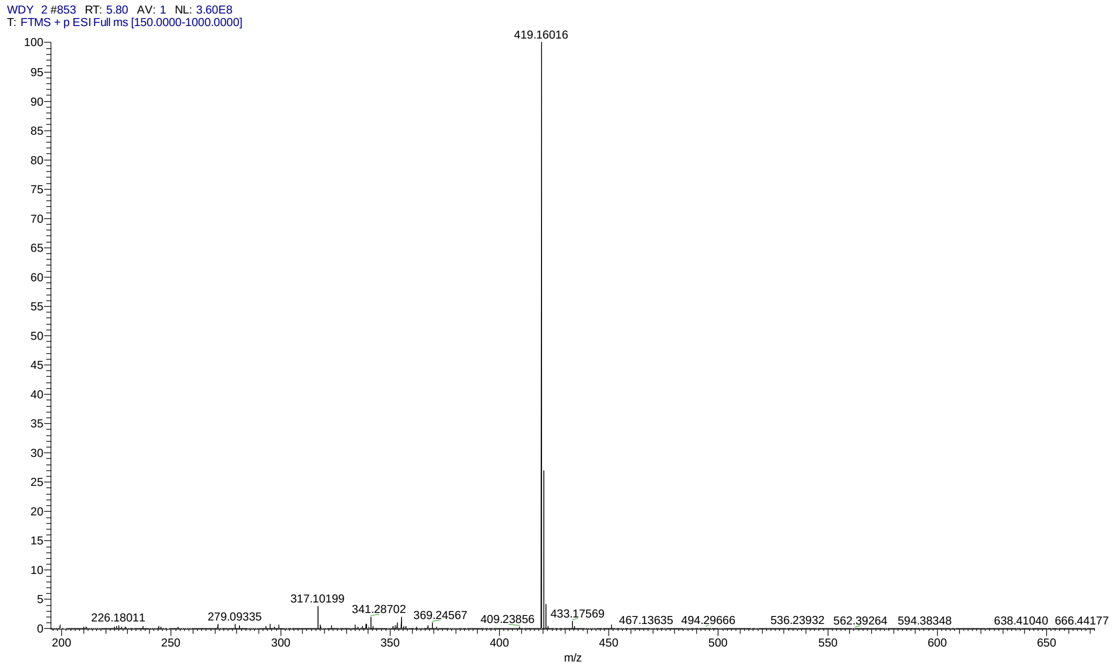

Figure S42. HRMS of Mollugin derivative **6e**

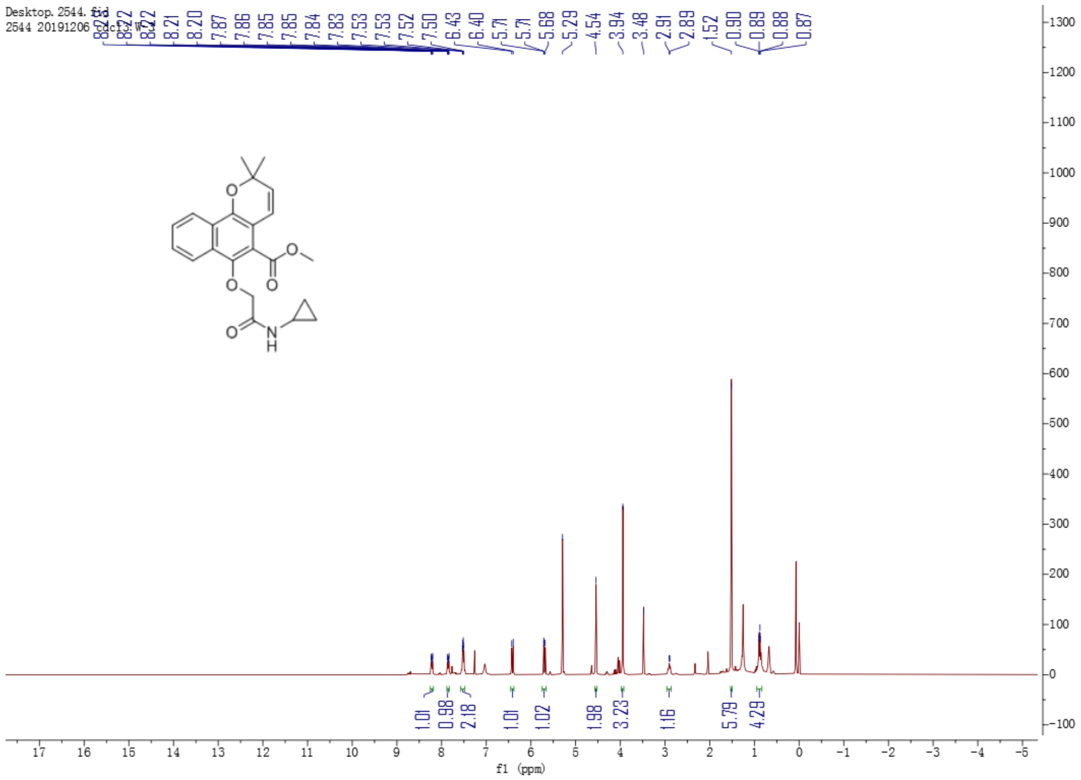

Figure S43. ^{1}H-NMR (300 MHz, $CDCl_3$) spectrum of Mollugin derivative **6f**

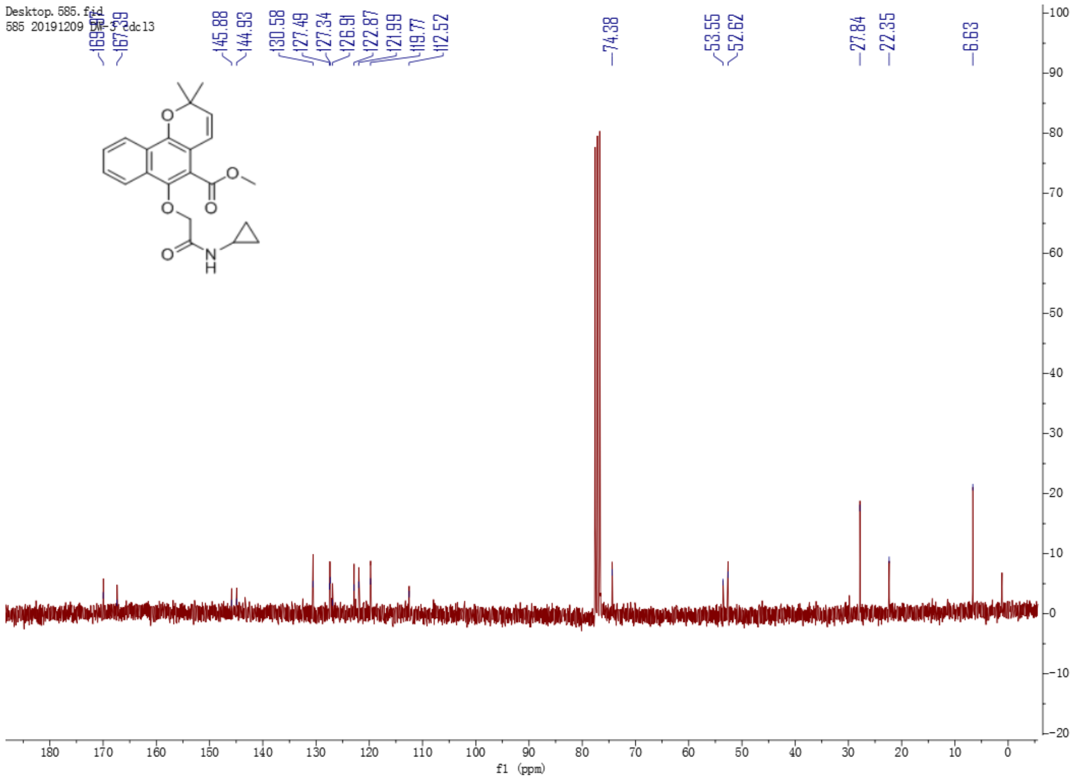

Figure S44. ^{13}C NMR (125 MHz, $CDCl_3$) spectrum of Mollugin derivative **6f**

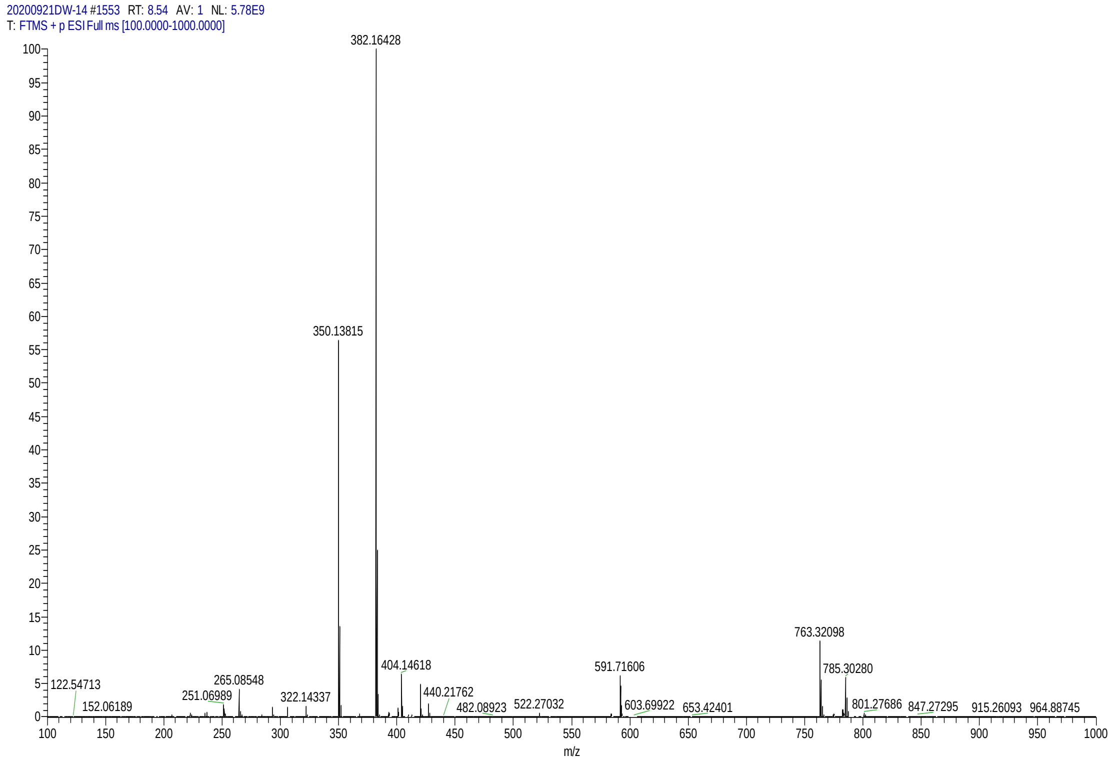

Figure S45. HRMS of Mollugin derivative **6f**

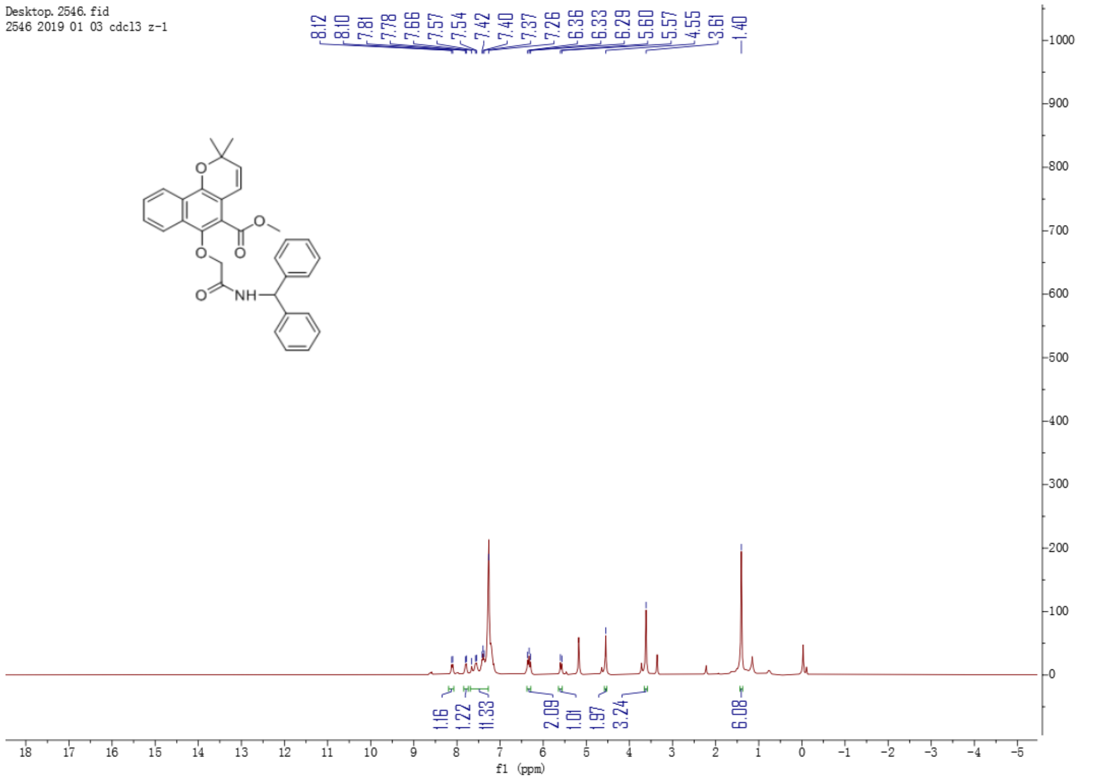

Figure S46. ^{1}H-NMR (300 MHz, $CDCl_3$) spectrum of Mollugin derivative **6g**

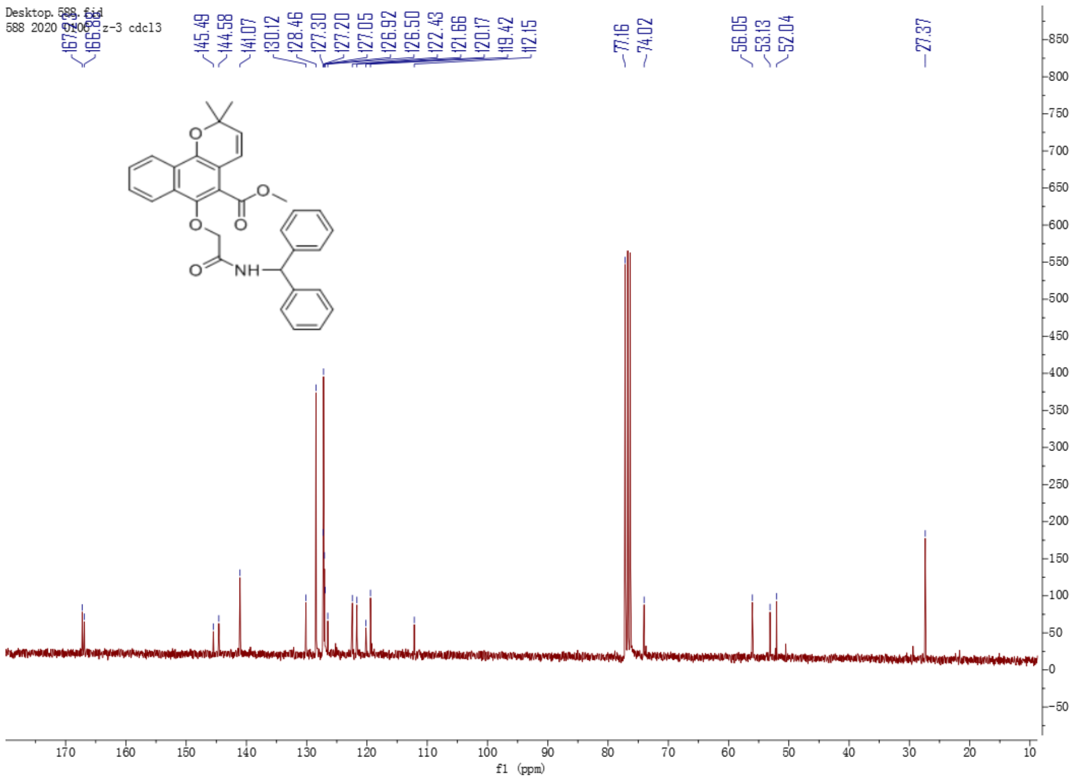

Figure S47. ^{13}C NMR (125 MHz, $CDCl_3$) spectrum of Mollugin derivative **6g**

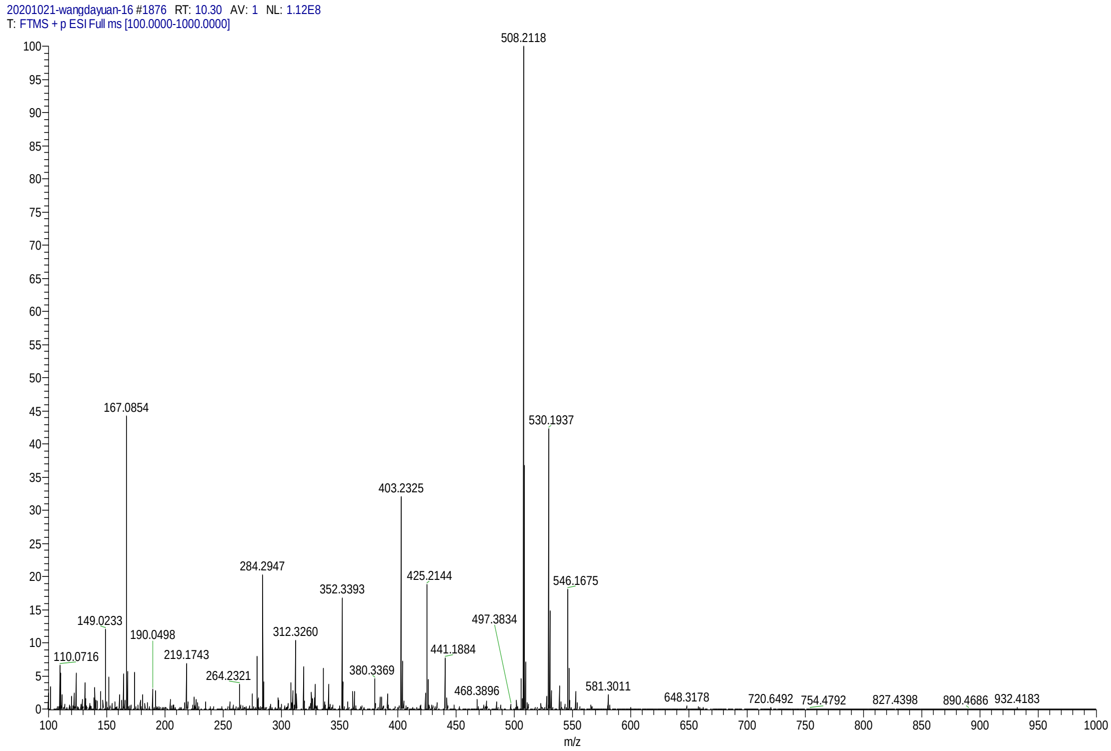

Figure S48. HRMS of Mollugin derivative **6g**

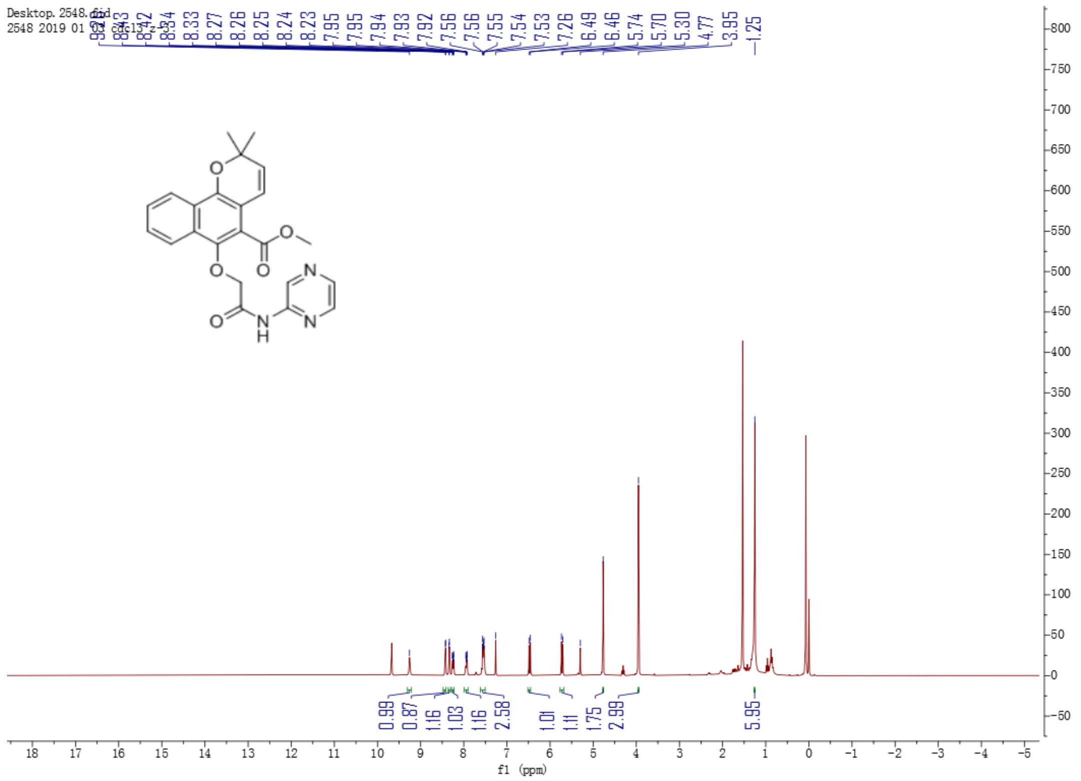

Figure S49. ^{1}H-NMR (300 MHz, $CDCl_3$) spectrum of Mollugin derivative **6h**

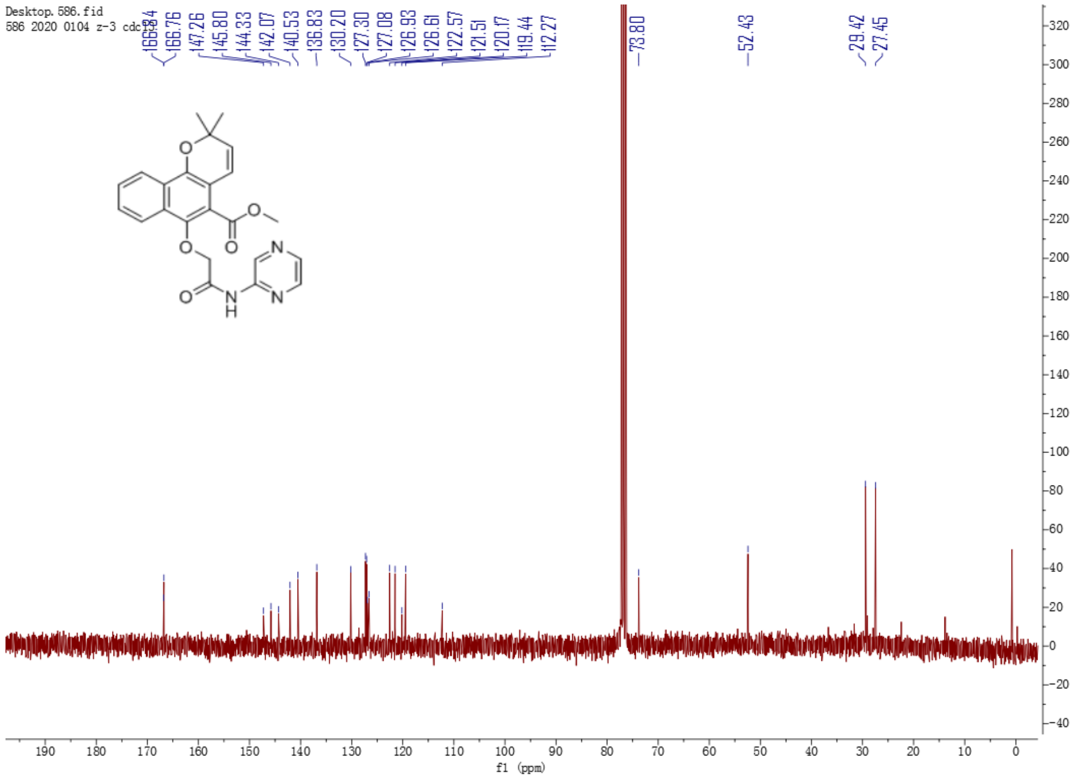

Figure S50. ^{13}C NMR (125 MHz, $CDCl_3$) spectrum of Mollugin derivative **6h**

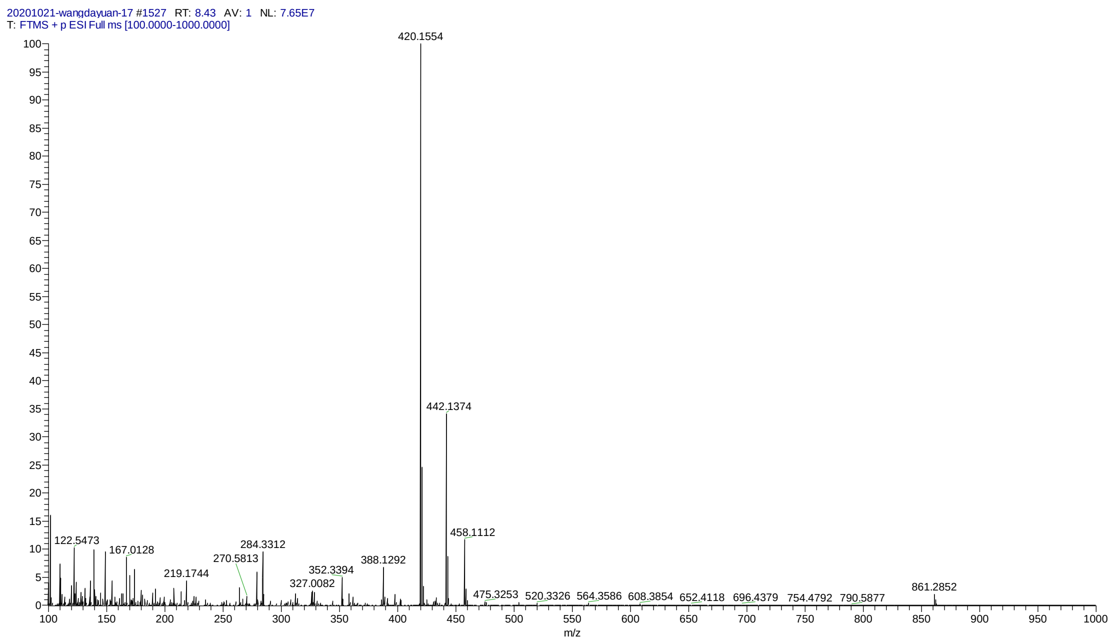

Figure S51. HRMS of Mollugin derivative **6h**

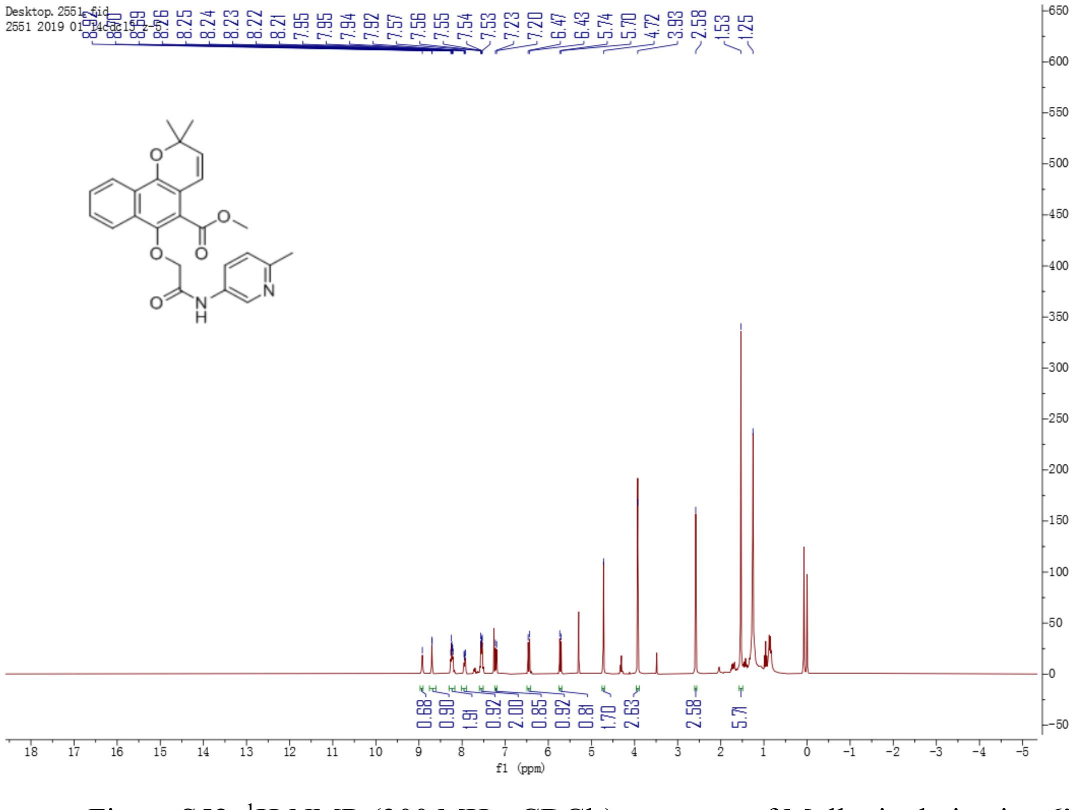

Figure S52. ^{1}H-NMR (300 MHz, $CDCl_3$) spectrum of Mollugin derivative **6i**

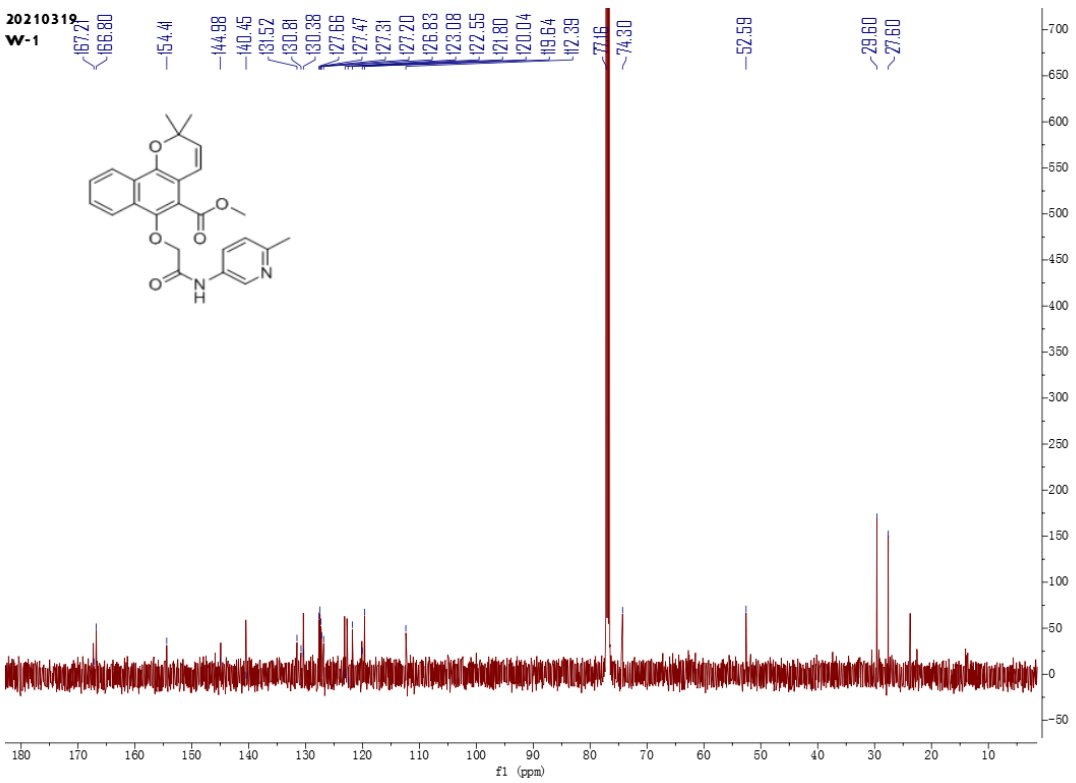

Figure S53. ^{13}C NMR (125 MHz, $CDCl_3$) spectrum of Mollugin derivative **6i**

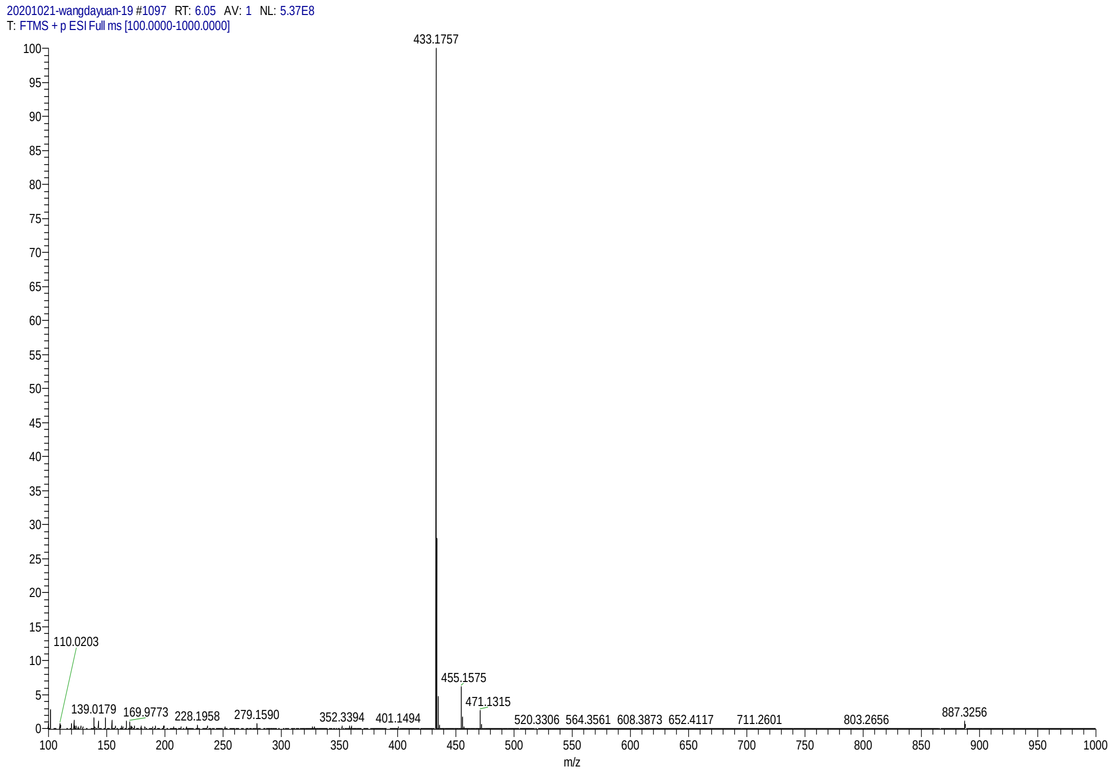

Figure S54. HRMS of Mollugin derivative **6i**

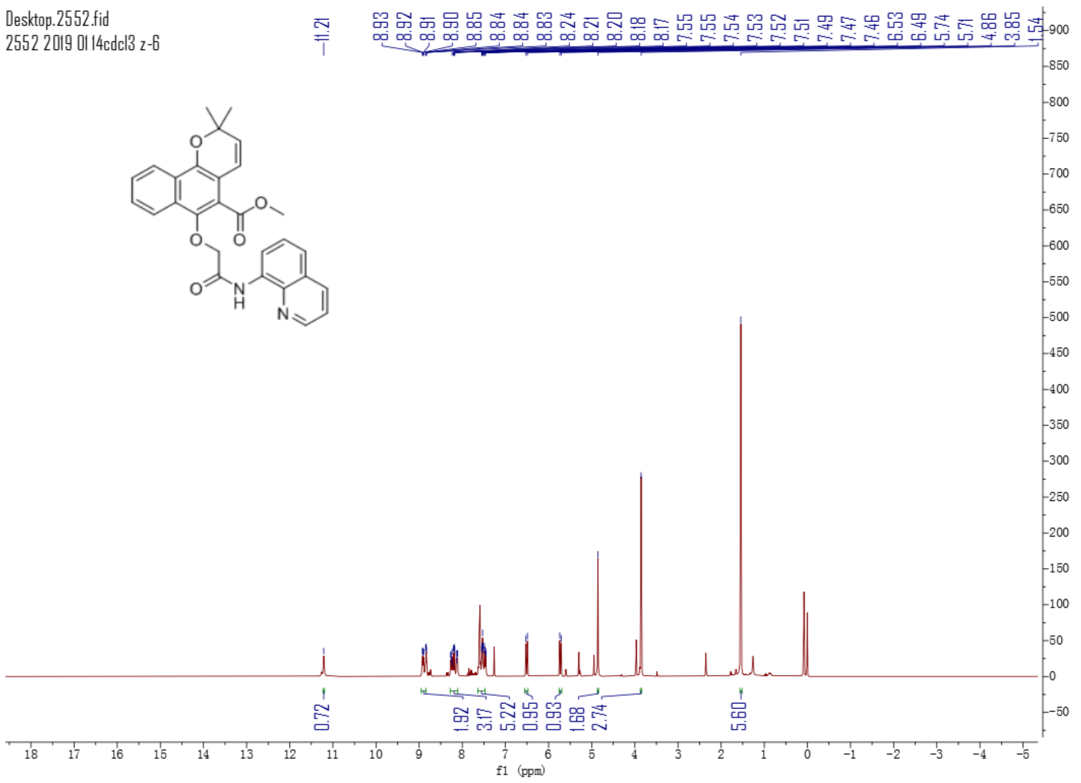

Figure S55. ^{1}H-NMR (300 MHz, $CDCl_3$) spectrum of Mollugin derivative **6j**

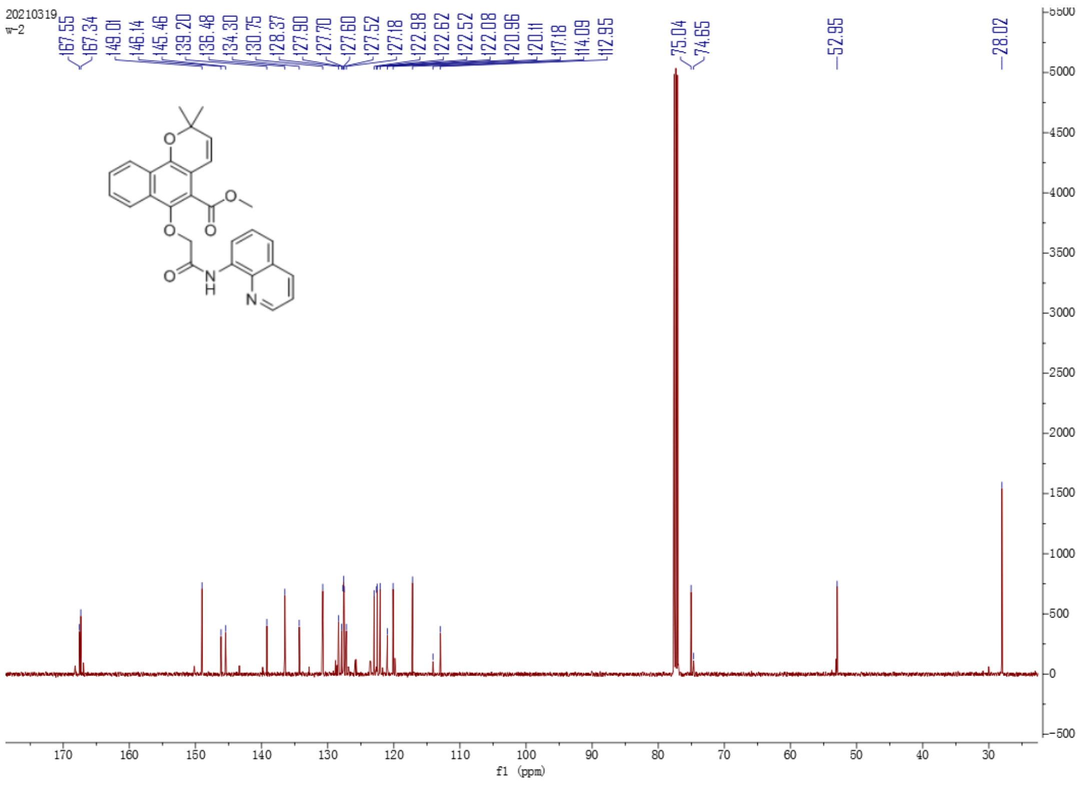

Figure S56. ^{13}C NMR (125 MHz, $CDCl_3$) spectrum of Mollugin derivative **6j**

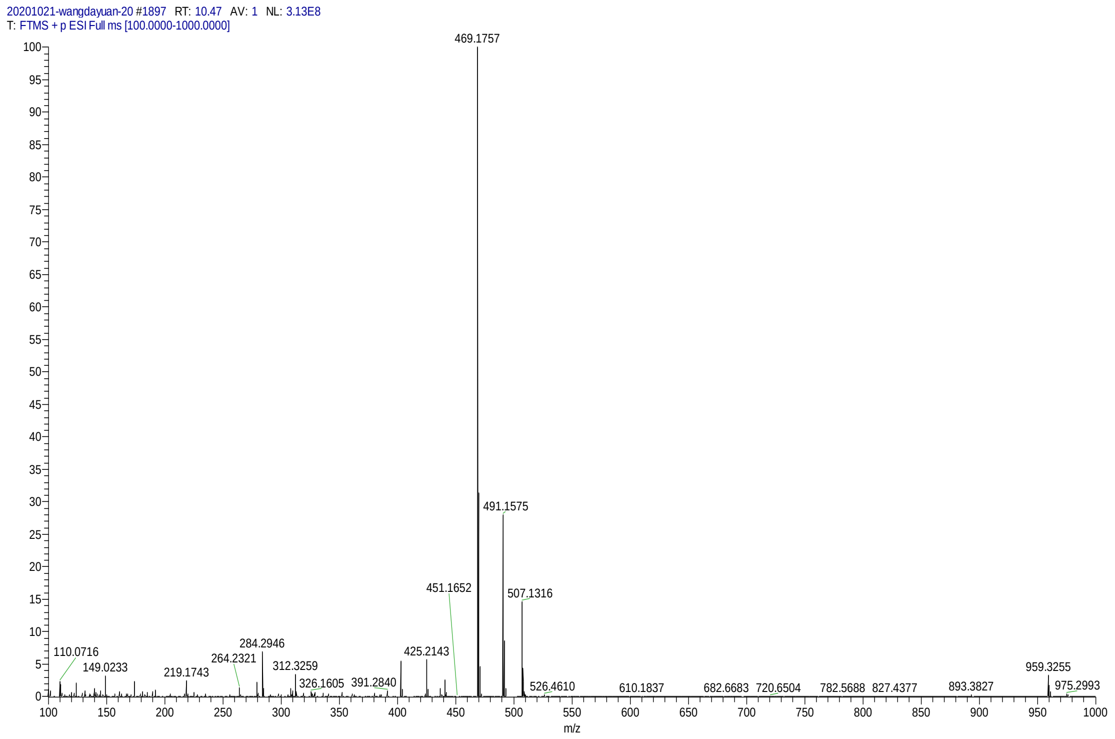

Figure S57. HRMS of Mollugin derivative **6j**

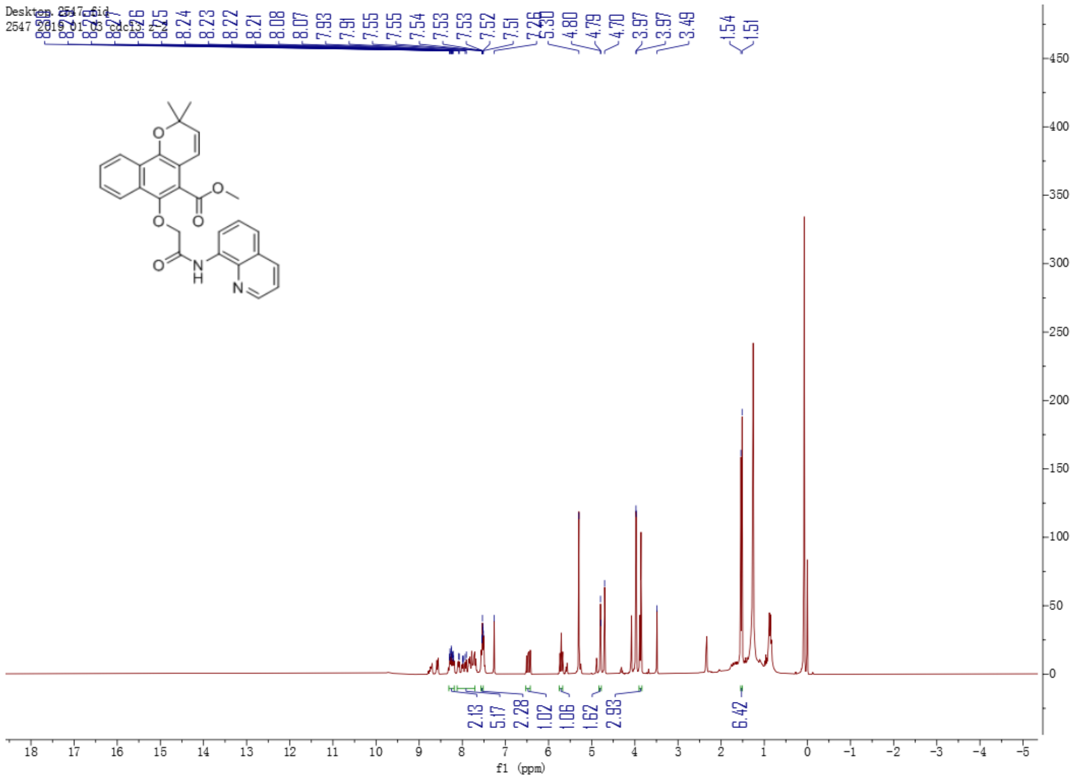

Figure S58. ^{1}H-NMR (300 MHz, $CDCl_3$) spectrum of Mollugin derivative **6k**

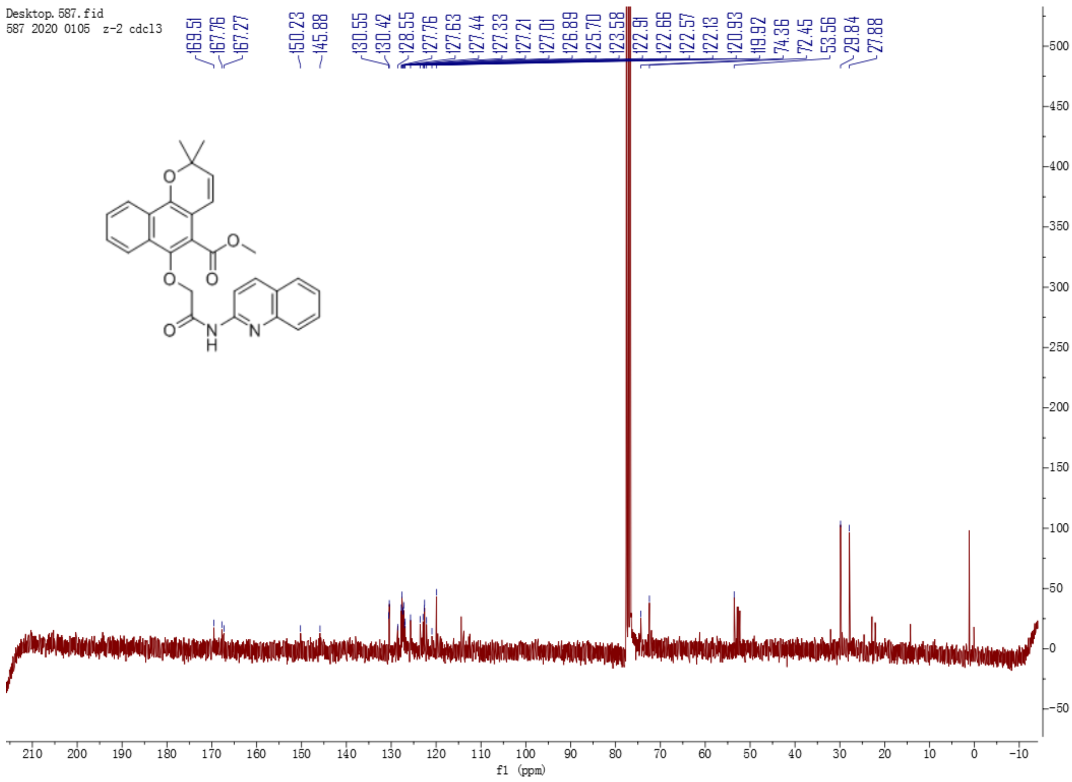

Figure S59. ^{13}C NMR (125 MHz, $CDCl_3$) spectrum of Mollugin derivative **6k**

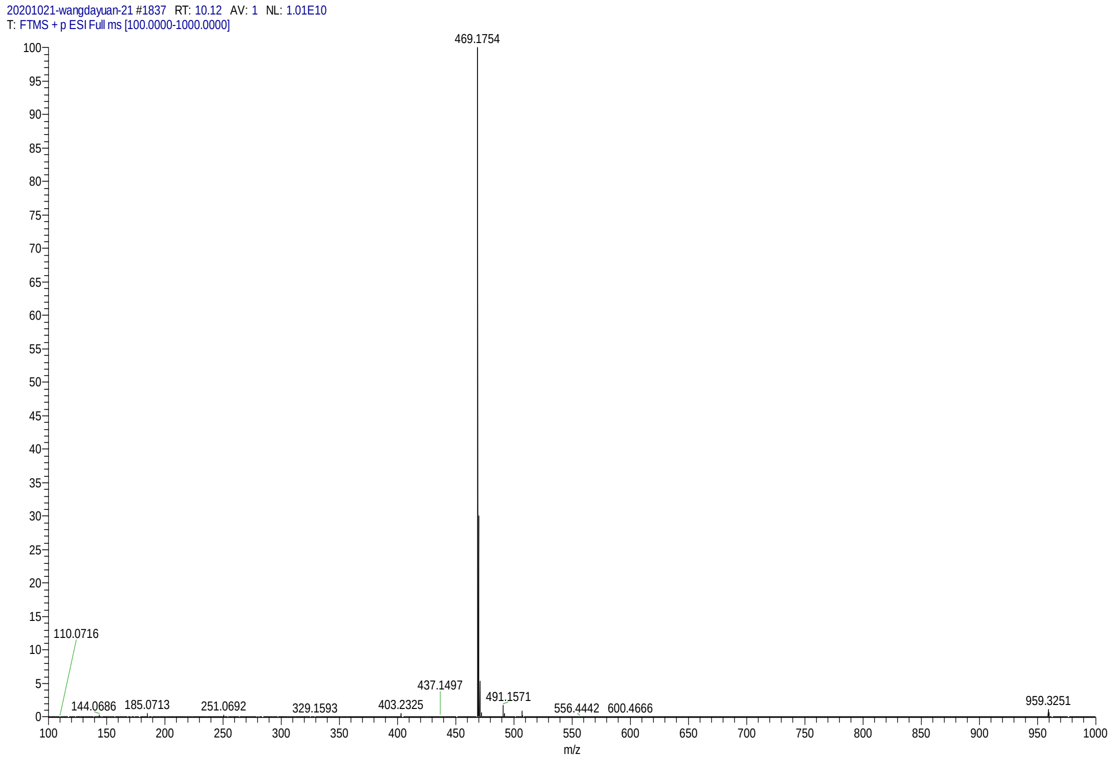

Figure S60. HRMS of Mollugin derivative **6k**

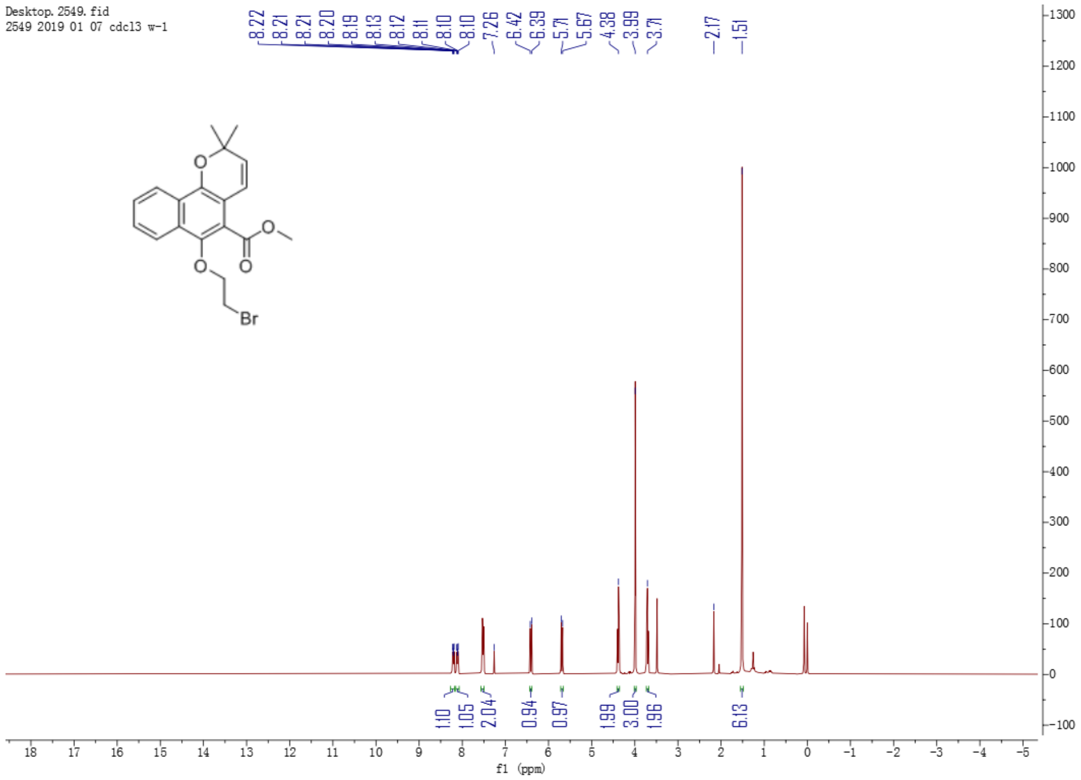

Figure S61. ^{1}H-NMR (300 MHz, $CDCl_3$) spectrum of Mollugin derivative **8a**

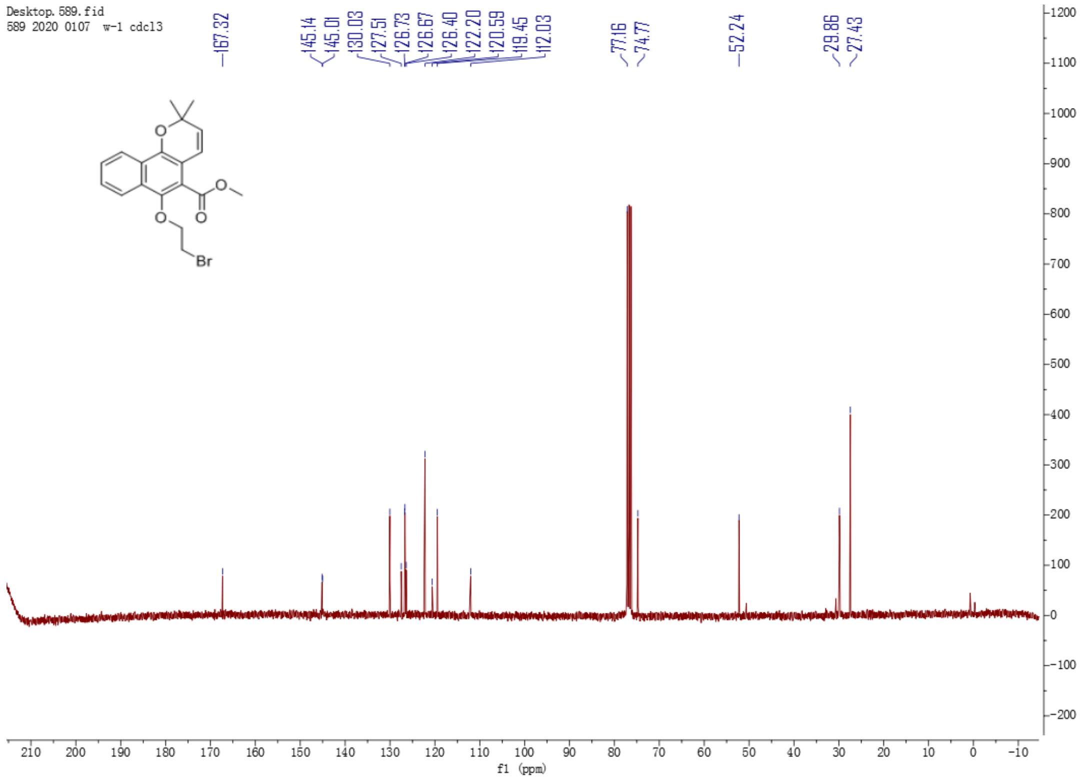

Figure S62. ^{13}C NMR (125 MHz, $CDCl_3$) spectrum of Mollugin derivative **8a**

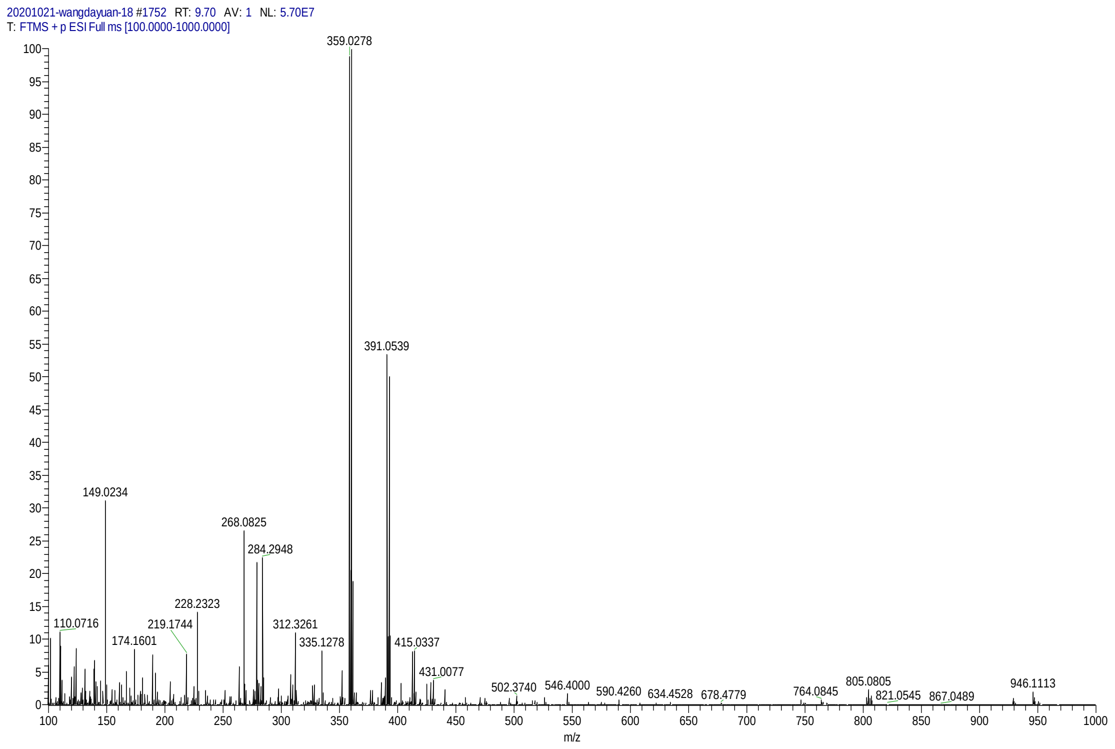

Figure S63. HRMS of Mollugin derivative **8**[a]

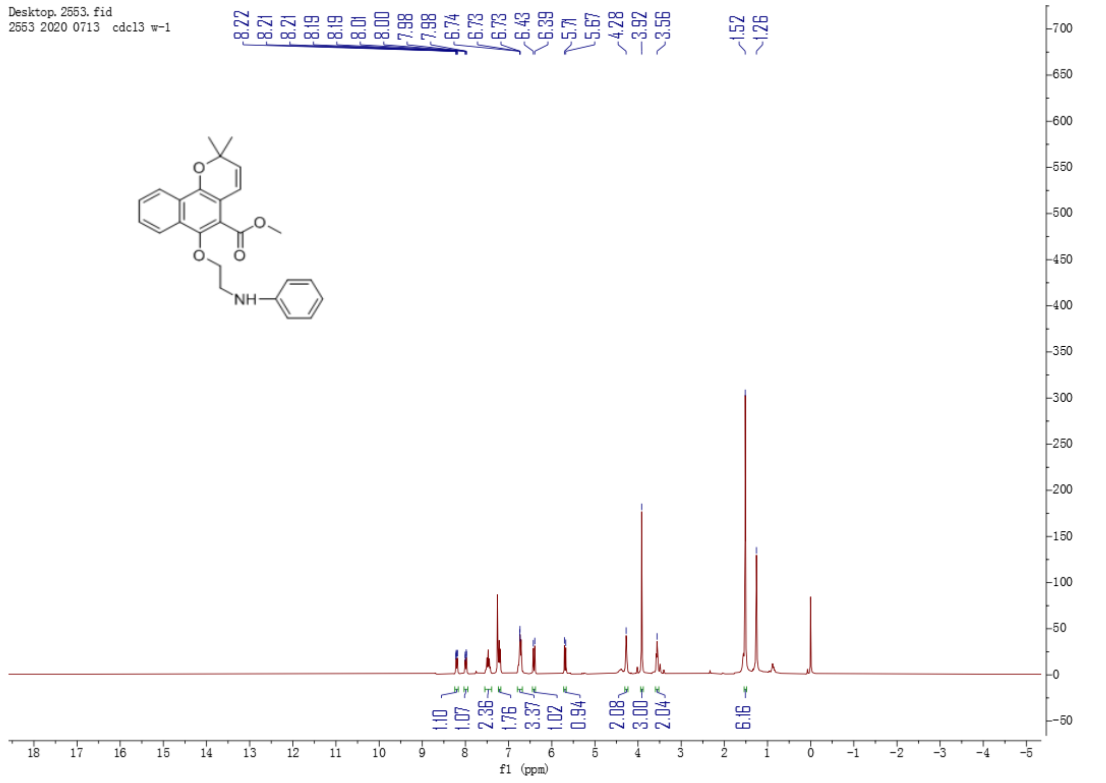

Figure S64. ^{1}H-NMR (300 MHz, $CDCl_3$) spectrum of Mollugin derivative **8b**

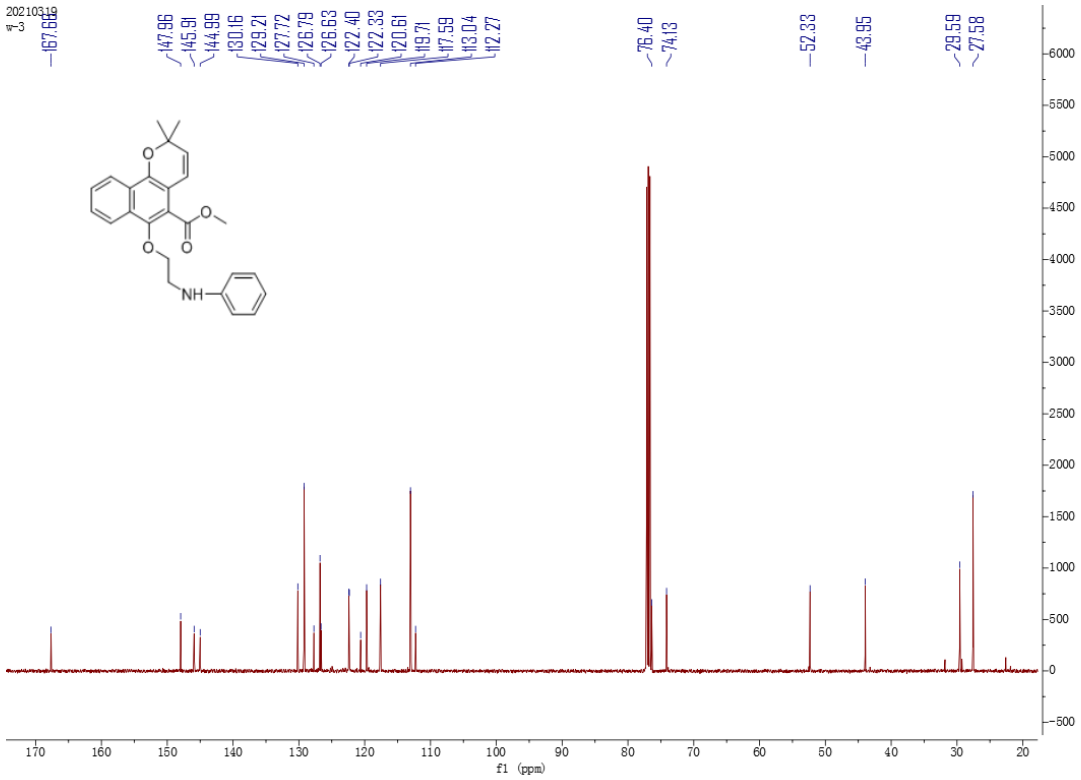

Figure S65. ^{13}C NMR (125 MHz, $CDCl_3$) spectrum of Mollugin derivative **8b**

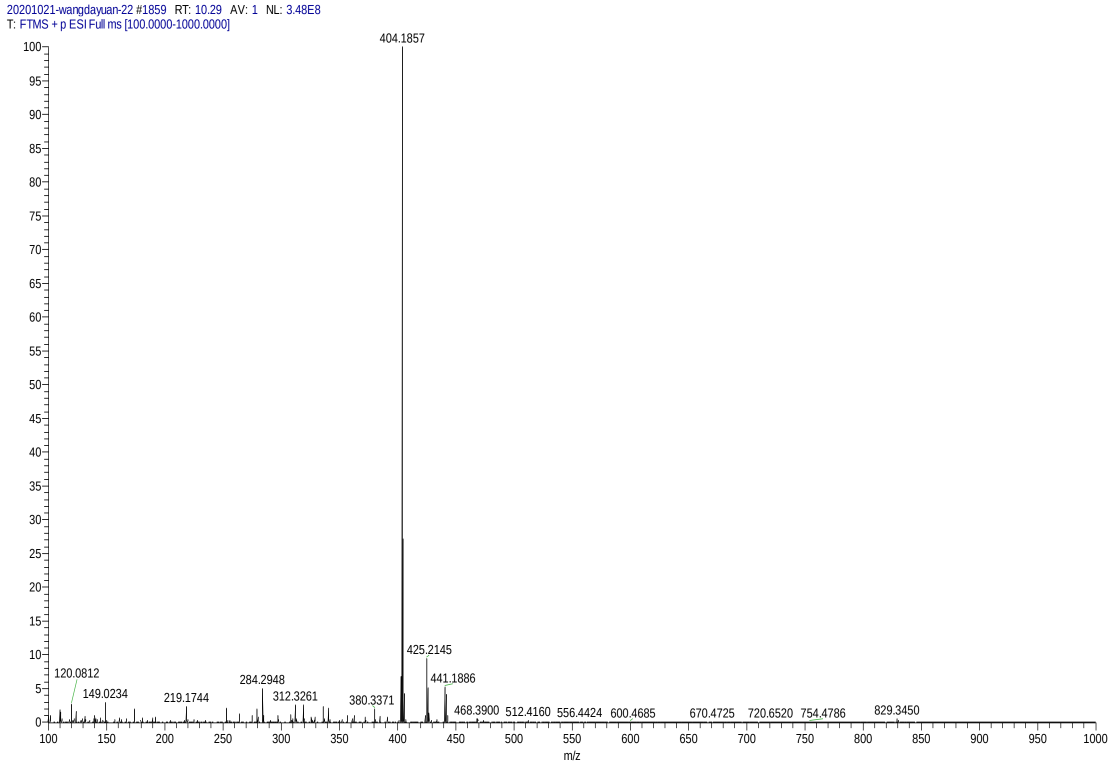

Figure S66. HRMS of Mollugin derivative **8b**

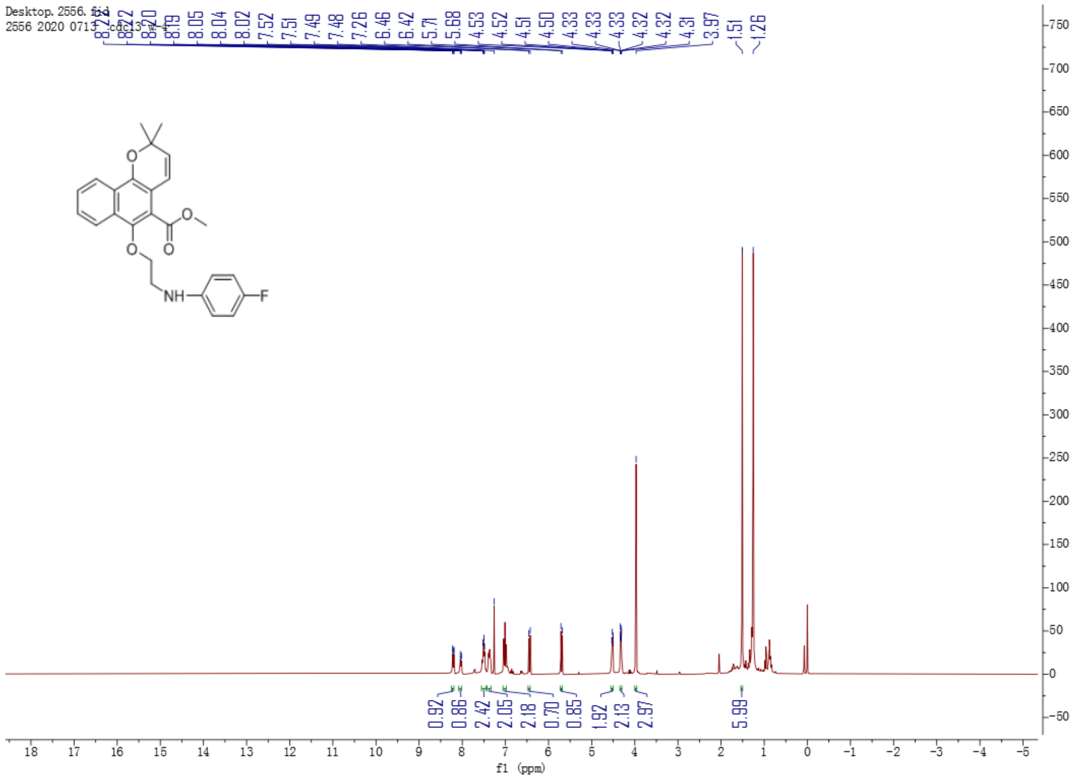

Figure S67. ^{1}H-NMR (300 MHz, $CDCl_3$) spectrum of Mollugin derivative **8c**

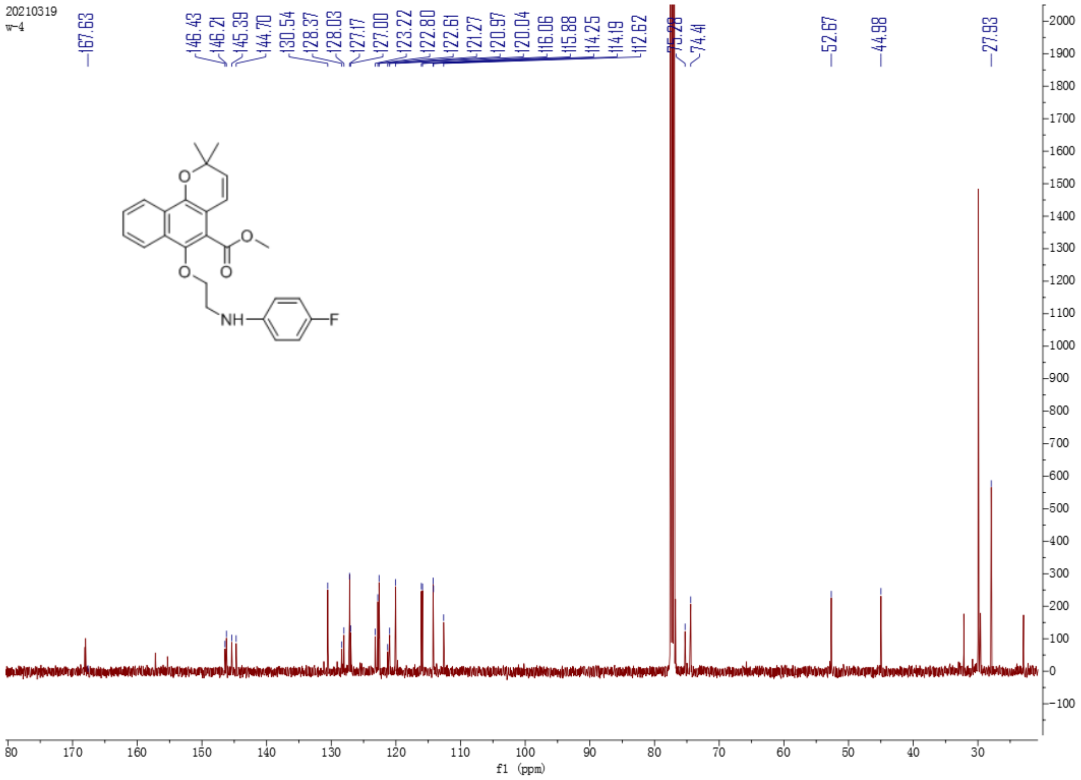

Figure S68. ^{13}C NMR (125 MHz, $CDCl_3$) spectrum of Mollugin derivative **8c**

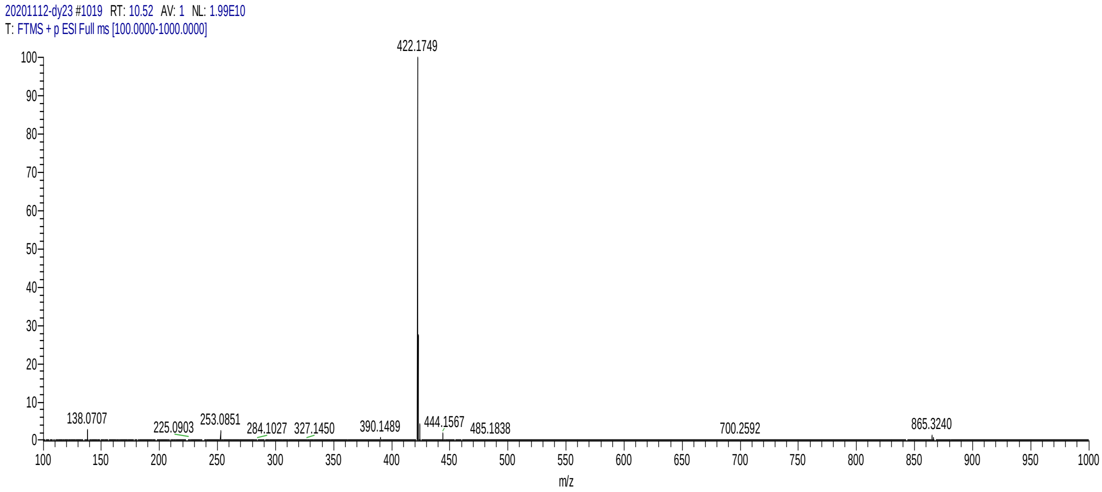

Figure S69. HRMS of Mollugin derivative **8c**
